# Transcriptomic analysis of pathways regulated by toll-like receptor 4 in a murine model of chronic pulmonary inflammation and carcinogenesis

**DOI:** 10.1186/1476-4598-8-107

**Published:** 2009-11-19

**Authors:** Alison K Bauer, Jennifer Fostel, Laura M Degraff, Elizabeth A Rondini, Christopher Walker, Sherry F Grissom, Julie Foley, Steven R Kleeberger

**Affiliations:** 1 Department of Pathobiology and Diagnostic Investigation, Michigan State University, East Lansing, MI, USA; 2 Laboratory of Respiratory Biology, National Institute of Environmental Health Sciences, Research Triangle Park, NC, USA; 3 Laboratory of Molecular Toxicology, National Institute of Environmental Health Sciences, Research Triangle Park, NC, USA; 4 Laboratory of Cellular and Molecular Pathology, National Institute of Environmental Health Sciences, Research Triangle Park, NC, USA

## Abstract

**Background:**

Therapeutic strategies exist for human pulmonary neoplasia, however due to the heterogeneity of the disease, most are not very effective. The innate immunity gene, toll-like receptor 4 (TLR4), protects against chronic pulmonary inflammation and tumorigenesis in mice, but the mechanism is unclear. This study was designed to identify TLR4-mediated gene expression pathways that may be used as prognostic indicators of susceptibility to lung tumorigenesis in mice and provide insight into the mechanism.

**Methods:**

Whole lung mRNA was isolated from C.C3H-*Tlr4*^*Lps*-*d *^(BALB^*Lps*-*d*^; *Tlr4 *mutant) and BALB/c (*Tlr4 *normal) mice following butylated hydroxytoluene (BHT)-treatment (four weekly ip. injections; 150-200 mg/kg/each; "promotion"). mRNA from micro-dissected tumors (adenomas) and adjacent uninvolved tissue from both strains were also compared 27 wks after a single carcinogen injection (3-methylcholanthrene (MCA), 10 μg/g; "control") or followed by BHT (6 weekly ip. injections; 125-200 mg/kg/each; "progression"). Bronchoalveolar lavage fluid was analyzed for inflammatory cell content and total protein determination, a marker of lung hyperpermeability; inflammation was also assessed using immunohistochemical staining for macrophages (F4/80) and lymphocytes (CD3) in mice bearing tumors (progression).

**Results:**

During promotion, the majority of genes identified in the BALB^*Lps*-*d *^compared to BALB/c mice (P < 0.05) were involved in epithelial growth factor receptor (EGFR) signaling (e.g. epiregulin (*Ereg*)), secreted phosphoprotein 1(*Spp1*)), which can lead to cell growth and eventual tumor development. Inflammation was significantly higher in BALB^*Lps*-*d *^compared to BALB/c mice during progression, similar to the observed response during tumor promotion in these strains. Increases in genes involved in signaling through the EGFR pathway (e.g. *Ereg*, *Spp1*) were also observed during progression in addition to continued inflammation, chemotactic, and immune response gene expression in the BALB^*Lps*-*d *^versus BALB/c mice (*P *< 0.05), which appears to provide more favorable conditions for cell growth and tumor development. In support of these findings, the BALB/c mice also had significantly reduced expression of many immune response and inflammatory genes in both the tumors and uninvolved tissue.

**Conclusion:**

This transcriptomic study determined the protective effect of TLR4 in lung carcinogenesis inhibition of multiple pathways including EGFR (e.g. *Ereg*), inflammatory response genes (e.g. *Cxcl5)*, chemotaxis (e.g. *Ccr1*) and other cell proliferation genes (e.g. *Arg1*, *Pthlh*). Future studies will determine the utility of these pathways as indicators of immune system deficiencies and tumorigenesis.

## Introduction

Adenocarcinoma (AC), a non-small cell lung carcinoma (NSCLC) [1], remains the leading type of lung cancer among smokers and nonsmokers. AC is often not detected until advanced stages of the disease thus making it the most clinically-intractable of lung cancers [1]. Therefore, it is critical to discover biomarkers of early stages of AC that would allow early detection and to find new sites at which chemopreventive agents could act to inhibit further neoplastic progression. Chronic inflammatory lung diseases, such as COPD and asthma, predispose to lung neoplasia [2-5]. Thus, inflammatory mediators and effector pathways might provide a source of early biomarkers and further insight into mechanisms of lung cancer.

Spontaneous or chemically-induced mouse lung neoplasms resemble those of human AC in anatomy, histogenesis, and molecular features [6], thereby facilitating mouse to human comparisons. Multiple exposures to butylated hydroxytoluene (BHT) elicit lung injury and inflammation during tumor promotion that are significantly correlated with tumor multiplicity in mice [7,8]. This 2-stage carcinogenesis model uses a low dose of a tobacco smokexs carcinogen, 3-methlycholanthrene (MCA), followed by multiple doses of BHT as the tumor promoter [9]. It is not BHT *per se*, but oxidative metabolites of BHT produced in high concentrations in mouse lung that are responsible for these pneumotoxicities and inflammatory activities [10]. Understanding the molecular events that occur early in the tumorigenic process (i.e. promotion including chronic inflammation and proliferation) may be critical to understanding the later events that occur during progression.

*Tlr4 *is an innate immunity gene involved in exacerbation of responses to several pulmonary agonists including endotoxin (lipopolysaccarride, LPS [11]) and ozone (O_3 _[12]), and injury protection from pulmonary insults, including hyperoxia [13] and allergic inflammation [14]. Several epidemiological studies found significant decreases in lung cancer risk in individuals such as farm and textile workers that were exposed to endotoxin [15-17]. Because TLR4 is the primary receptor that binds endotoxin [11], it is likely that TLR4 is involved in protection observed with endotoxin exposure. TLR4 also confers protection against human gastric and mouse cutaneous carcinomas [18,19]. The mechanism behind TLR4-mediated protection is unclear.

We previously demonstrated that TLR4 protects against BHT-induced chronic pulmonary inflammation and tumor promotion [20]. C.C3H-*Tlr4*^*Lps*-*d *^(BALB^*Lps*-*d*^) mice have a missense mutation in *Tlr4 *[21] that renders TLR4 dysfunctional. Bronchoalveolar lavage fluid (BALF) inflammatory markers were significantly elevated in BALB^*Lps*-*d *^mice compared to BALB/c (BALB; *Tlr4 *wild type) mice following BHT treatment [20]. Significantly increased tumor multiplicity (60%) was also found in BALB^*Lps*-*d *^compared to BALB mice in response to MCA/BHT induced tumor promotion. However, the downstream mechanism regulating this protective response remains unknown.

In the present investigation, we hypothesized that gene transcripts that are highly correlated to TLR4 associate with differential susceptibility to an early stage (BHT-treatment) and a later stage (progression) of lung tumorigenesis. To test this hypothesis we used two methods to analyze transcriptome responses in lung tissue from BALB and BALB^*Lps*-*d *^mice during promotion, and in tumors and uninvolved lung tissue in advanced stages of tumorigenesis. The first statistical method (supervised) assigned a significance value to transcript changes. The second method (unsupervised, pattern analysis) used *k*-means clustering to identify additional patterns which may have remained hidden. These methods identified changes in transcripts known to be downstream of TLR4 and others not previously linked to this gene. We suggest that these pathways and interactions amongst the genes identified during tumor promotion influence the TLR4-mediated response observed during progression of tumorigenesis.

## Methods

### Animals

Male 5-7 wk. old BALB/c (BALB; *Tlr4 *wild type) and C.C3-*Tlr4*^*Lps*-*d*^/J (BALB^*Lps*-*d*^; *Tlr4 *dominant negative) mice were purchased from Jackson Laboratories (Bar Harbor, ME). Mice acclimated for 1 wk prior to treatment. All animal use was conducted in facilities accredited by the Association for the Assessment and Accreditation of Laboratory Animal Care and approved by the NIEHS Animal Care and Use Committee and the MSU Institutional Animal Care and Use Committee. Mice were housed in shoebox cages in a humidity and temperature-controlled room and provided water and pelleted open-formula rodent diet NIH-07 (Zeigler Brothers, Gardners, PA.) *ad libitum*.

### Experimental design

The two protocols used for these studies (see Additional file 1, Figure S1) were based on previous studies demonstrating a correlation between chronic inflammation and tumor promotion [7]. To induce inflammation (Protocol 1: promotion; Additional file 1, Figure S1: A), mice were injected intraperitoneally (ip.) with BHT (Sigma, St. Louis, MO) weekly for 4 weeks (150 mg/kg for the first dose followed by 200 mg/kg for the next 3 doses). Mice were then sacrificed 1 or 3 days following the last BHT dose. For BHT-induced tumor studies (Protocol 2: progression stage; Additional file 1: Figure S1: B), 10 μg MCA/gm (Sigma) body wt. was injected ip., followed by 150 mg BHT/kg or corn oil vehicle control one week later. Five additional weekly 200 mg/kg BHT doses or vehicle control doses were administered to maximize tumor promotion [22]. Mice were sacrificed 27 wks after MCA injection. Because fewer than 0.5 tumors/mouse result from MCA alone, the tumors that develop after MCA/BHT treatment are primarily those promoted by MCA.

The left lung of each mouse was clamped off at the mainstem bronchus and the right lung was lavaged for inflammatory infiltrate analysis [12,20]. Briefly, four aliquots of HBSS (Sigma) were inserted sequentially into the lung based on body weight (17.5 ml/kg). The first lavage was used for protein analysis (BioRad protein assay, BioRad, Hercules, CA) as a measure of lung hyperpermeability. Total pooled cells from the four lavages were then counted, followed by cytocentrifugation (Shandon Southern Products, Pittsburgh, PA). The slides were then stained with a modified Wright's stain (Hema 3 Stain Set, Fisher Scientific, Pittsburgh, PA) to differentiate inflammatory cells by morphology, including PMNs, alveolar macrophages, lymphocytes, and epithelial cells. The left lung was snap frozen in liquid nitrogen. For MCA/BHT treated mice (Protocol 2), the lavaged right lung was inflation-fixed with 10% formalin, processed, and embedded. Tumors (adenomas) were carefully micro-dissected away from the adjacent tissue on left lung lobe using a Leica S6D stereo microscope (Leica Microsystems, Inc., Bannockburn, IL) and then snap frozen, similar to other studies [23-25]. Remaining whole lung tissue was used as the uninvolved tissue. In addition, whole lung tissue was also used for the MCA/oil treatment group. Due to the manner in which the tumors were micro-dissected away from the normal adjacent uninvolved tissue, there is a possibility that the uninvolved tissue may contain some remaining smaller lesions as well as tumor cells from the periphery.

### Immunohistochemistry for CD3 and F4/80

5 μm thick sections were stained using antibodies for CD3 (ab5690; Abcam, Cambridge, MA) as a pan-T-lymphocyte marker and F4/80 (MF48000; Caltag, Burlingame, CA) as a macrophage marker, following standard protocols at: http://www.niehs.nih.gov/research/atniehs/labs/lep/path-support/immuno/.

### Total lung RNA isolation and cDNA synthesis for mRNA analyses

Total RNA was isolated from the left lung lobe using RNeasy Mini Kits (Qiagen, Valencia, CA) following kit specifications, including DNase 1 treatment. cDNA was synthesized as described previously [26]. For Protocol 1, RNA was isolated from each of three replicate mice for each experimental group (experimental groups = oil control, 1-day, and 3-days after BHT, three replicated per group). For Protocol 2, tumors from 2 to 3 mice were pooled to obtain sufficient material; uninvolved tissue from the same mice was also pooled for control. There were two replicates for each experimental group (MCA/oil, MCA/BHT tumor tissue and MCA/BHT uninvolved tissue) except for the BALB tumor group where the incidence of tumors was low.

### Affymetrix Mouse 430A_MOE array analysis

Total RNA was used only after it passed quality testing performed using a 2100 Bioanalyzer (Agilent Technologies, Inc., Santa Clara, CA). Gene expression analysis was conducted using Affymetrix MOE430A or MOE430Av2 GeneChip^® ^arrays (Affymetrix, Santa Clara, CA) as recommended by the manufacturer. Total RNA (1 μg) was amplified using the Affymetrix One-Cycle cDNA Synthesis protocol. For each array, 15 μg of amplified biotin-cRNAs was fragmented and hybridized to the array for 16 hours at 45°C in a rotating hybridization oven using the Affymetrix Eukaryotic Target Hybridization Controls and protocol. Slides were stained with steptavidin/phycoerythrin using a double-antibody staining procedure and washed utilizing the Mini_euk2v3 Protocol of the Affymetrix Fluidics Station FS450 for antibody amplification. Arrays were scanned with an Affymetrix Scanner 3000 and data obtained using the GeneChip^® ^Operating Software.

The CEL files for each array type were normalized and genes with altered expression level were selected using k-means clustering and ANOVA (p < 0.05). The raw data discussed in this publication have been deposited into NCBI Gene Expression Omnibus (GEO, http://www.ncbi.nlm.nih.gov/geo/, Series GSE8504) and NIEHS Chemical Effects in Biological Systems (CEBS, http://cebs.niehs.nih.gov/, accession 005-00003-0030-000-1). Starting and ending gene lists are found in Additional file 2, Table S1. The details of the process to identify genes with altered transcript levels using the supervised and unsupervised methods are found in Additional file 3.

### Quantitative real time PCR (qRTPCR)

qRTPCR was performed using Sybr green assays on an Applied Biosystems 7900 Prism Sequence Detection System as follows: 12.5 μl Power Sybr Green PCR master mix (Applied Biosystems, Foster City, CA), 200 nM forward and reverse primers, 5.5 μl nuclease-free dH_2_O, and 2 μl of cDNA per 25 μl reaction. The reverse and forward primers can be found in Additional file 4, Table S2. PCR reaction conditions and data analysis were performed according to the manufacturer's protocol (User bulletin no.2, Applied Biosystems Prism 7700 Sequence Detection System). 18S was used to normalize all genes of interest since it did not vary across genotypes or treatments. Some transcript changes identified by microarray analysis were confirmed using qRTPCR in the same samples (Protocol 1) and in the same samples plus samples from a parallel study (Protocol 2).

### Statistics for BALF analysis and qRTPCR

Data are expressed as the group mean ± SEM. Two-way ANOVA was used to evaluate the effects of strain (BALB vs. BALB^*Lps*-*d*^) and treatment (MCA/oil and MCA/BHT) on BAL phenotypes. A 3-way ANOVA was performed for treatment, time (0, 1, or 3 days), and strain effects on mRNA expression data under Protocol 1 (promotion). A 2-way ANOVA was performed for the treatment (MCA/oil, MCA/BHT tumor tissue, and MCA/BHT uninvolved tissue) and strain effects on mRNA expression data under Protocol 2 (progression). Student-Newman-Keuls test was used for *a posteriori *comparisons of means. All analyses were performed using a commercial statistical analysis package (SigmaStat; Jandel Scientific Software, San Rafael, CA). Statistical significance was accepted at *P *< 0.05.

## Results

### BHT-induced transcriptome changes between BALB vs. BALB^*Lps*-*d *^mice during tumor promotion (Protocol 1, "Promotion")

**Figure 1:**
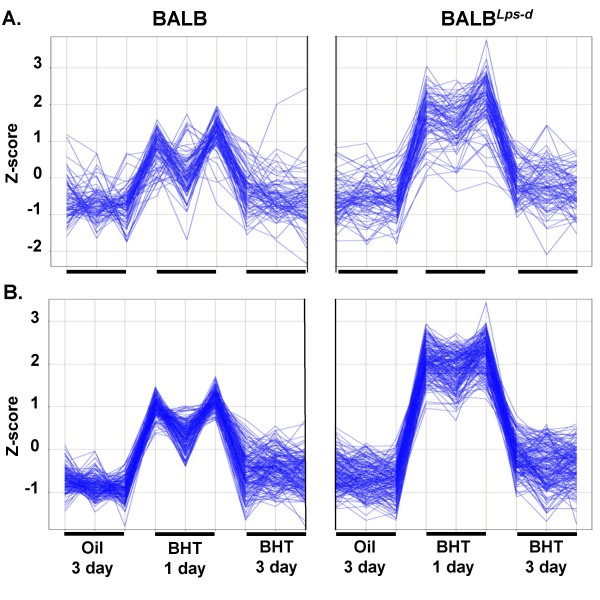
**Gene expression that increased in the BALB^*Lps*-*d *^mice compared to BALB mice following chronic BHT**. Two methods of analysis, supervised and unsupervised, were used to determine the genes increased at 1 day following BHT. A. Cluster 3 identified using statistical (GeneSpring) analysis in chronic BHT-treated BALB and BALB^*Lps*-*d *^mice over a time course (oil and 1 and 3 day following chronic BHT). Each vertical line represents one sample for each strain for an n = 3 (oil, BHT 1 day, BHT 3 day), indicated by the small horizontal black line above each treatment on the x-axis. B. Profile analysis (Spotfire) similar to that of cluster 3. Each vertical line represents one sample for each strain for an n = 3 (oil, BHT 1 day, BHT 3 day). Z-score (y-axis) is the intensity in terms of standard deviations (SD) from the mean. The z-score was calculated by subtracting the mean and dividing by the SD for each probeset. See Additional file 5, Figure S2 for supervised and unsupervised analyses depicted as a heat map.

Three different gene expression profiles were identified (genes for each are in Additional file 2, Table S1, Tab A). Figure 1 and Additional file 5, Figure S2 illustrate one of the profiles found by supervised (Fig 1A) and unsupervised (Fig 1B) methods. The profiles are biologically similar, i.e. expression increased by one standard deviation (BALB) or more (BALB^*Lps*-*d*^) in mice sampled one day following the last BHT injection compared to mice three days after BHT, or mice receiving oil vehicle control. We termed this profile "BHT_1day_up". Some genes with this profile were identified by both methods, and others found by only one method. We also found two BHT_1day_down profiles by supervised and unsupervised methods (see Additional file 2, Table S1, Tab A).

Transcripts with the BHT_1day_up profile have Gene Ontology (GO) functions involving cell cycle and growth, transcription, cell shape and morphogenesis, protein synthesis, protein targeting, and inflammatory and immune response (Table 1). Transcripts with the BHT_1day_down profile have functions related to cell cycle and growth, cell shape, protein targeting, and transcription (Table 2). The BHT_1day_up profile also includes genes with inflammation and immune response functions, as would be expected based on the BALF inflammation cell analysis from the same animals (data not shown).

**Table 1:** Representative gene expression changes increased in BALBLsp-d compared to BALB mice.


**Gene Symbol**	**Gene Name**	**Affymetrix Probe ID**	**Major GO Category(ies)**

**Angiogenesis**			
*Hbegf*^†^	heparin-binding EGF-like growth factor	1418349_at	angiogenesis; blastocyst growth
*Mmp19*^†^	matrix metallopeptidase 19	1421977_at	angiogenesis; peptidoglycan metabolic process
*Timp1*^†^	tissue inhibitor of metallo-proteinase 1	1460227_at	erythrocyte maturation
			
**Cell cycle and cell growth**			
*Cdkn1a*^†^	cyclin-dependent kinase inhibitor 1A (P21)	1424638_at	regulation of progression through cell cycle; response to DNA damage stimulus
***Ereg*^†‡^**	epiregulin	1419431_at	regulation of progression through cell cycle; angiogenesis
*Mina*	myc induced nuclear antigen	1451042_a_at	regulation of cell proliferation
*Myc*^†^	myelocytomatosis oncogene	1424942_a_at	regulation of progression through cell cycle; release of cytochrome c from mitochondria
*Socs3*	suppressor of cytokine signaling 3	1455899_x_at	regulation of cell growth; regulation of protein amino acid phosphorylation
			
**Cell shape and morphogenesis**			
*Krt19*	keratin 19	1417156_at	cytoskeleton organization and biogenesis
*Tnc*^†^	tenascin C	1416342_at	cell adhesion
*Tuba1c*	tubulin, alpha 1C	1416128_at	microtubule-based process and movement
			
**Inflammatory and immune response**			
*Il6*^†^	interleukin 6	1450297_at	neutrophil apoptosis
*Lif*^†^	leukemia inhibitory factor	1421207_at	immune response; tyrosine phosphorylation of stat3 protein
*Spp1*^†^	secreted phosphoprotein 1	1449254_at	ossification; anti-apoptosis
			
**Protein synthesis and turnover**			
*Eif1a*	eukaryotic translation initiation factor 1A	1419736_a_at	translation and initiation
*Hras1*^†^	Harvey rat sarcoma virus oncogene 1	1422407_s_at	translation; endocytosis
*Nola1*	nucleolar protein family A, member 1	1418305_s_at	rRNA processing; ribosome biogenesis and assembly
*Nola2*	nucleolar protein family A, member 2	1416605_at	rRNA processing; translation
*Nol5A*	nucleolar protein 5	1450986_at	ribosome biogenesis and assembly
*Nmd3*	NMD3 homolog	1437238_x_at	ribosomal large subunit export from nucleus
*Rps6ka3*	ribosomal protein S6 kinase polypeptide 3	1427299_at	protein amino acid phosphorylation; ribosome biogenesis and assembly
			
**Protein targeting**			
*Afp*^†^	alpha fetoprotein	1416646_at	ovulation; transport
*Bcl3*^†^	B-cell leukemia/lymphoma 3	1418133_at	protein import into nucleus, translocation; follicular dendritic cell differentiation
*Bnip1*	BCL2/adenovirus E1B interacting protein 1, NIP1	1427908_at	ER to Golgi vesicle-mediated transport; apoptosis
*Nup62*	Nucleoporin 62	1438917_x_at	protein targeting; transport
			
**Transcription, processing, and chromatin structure**			
**Bhlhb8**	basic helix-loop-helix domain containing, class B, 8	1449233_at	regulation of transcription, DNA-dependent
**Ddx39**	DEAD (Asp-Glu-Ala-Asp) box polypeptide 39	1423643_at	mRNA processing; RNA splicing
*Hmga1*^†^	high mobility group AT-hook 1	1416184_s_at	DNA packaging; transcription
**Lmna**	lamin A	1425472_a_at	nuclear membrane organization and biogenesis
*Pparg*^†^	peroxisome proliferator activated receptor gamma	1420715_a_at	negative regulation of transcription from RNA polymerase II promoter

These genes were identified in profile "BHT-1day_up" (A) and profile "BHT-1day_down" (B) below the p value set for the DAVID analysis (p < 0.0003)*; see Additional file 2, Table S1 for the complete profile gene lists.

*These genes were identified after bioinformatic analysis filtering first on biological patterns using both Genespring and Spotfire Software followed by filtering on GO biological categories using the DAVID Bioinformatics Database. ^†^These genes were upregulated ≥ 2-fold in BALB^*Lsp*-*d *^vs. BALB 1 day following protocol 1 (chronic BHT) (P < 0.05). ^‡^Significant interaction effects between strain (BALB vs BALB^*Lpsd *^mice) and treatment (oil, 1 or 3 days following 4BHT) (p < 0.05). Bolded genes are those genes in common with the gene list identified for Protocol 2 (see Table 2).

**Table 2:** Representative gene expression changes decreased in BALBLsp-d compared to BALB mice.

**B**.			
Gene Symbol*	Gene Name	Affymetrix Probe ID	Major GO Category(ies)
**Cell cycle and cell growth**			
*Csf1r*	colony stimulating factor 1 receptor	1419872_at	regulation of progression through cell cycle; protein amino acid phosphorylation
*Fgf7*^†^	fibroblast growth factor 7	1422243_at	regulation of progression through cell cycle;; signal transduction
*Gas6*	growth arrest specific 6	1417399_at	regulation of cell growth
*Htra1*	htrA serine peptidase 1	1416749_at	regulation of cell growth; proteolysis
*Itm2b*	integral membrane protein 2B	1418000_a_at	apoptosis
			
**Cell shape and morphogenesis**			
*Itga8*^†^	integrin alpha 8	1427489_at	cell adhesion
*Nox4*^†^	NADPH oxidase 4	1419161_a_at	cell morphogenesis; electron transport
*Pik3r1*^†^	phosphatidylinositol 3-kinase, regulatory subunit, polypeptide 1 (p85 alpha)	1425515_at	negative regulation of cell-matrix adhesion; protein amino acid phosphorylation
*Postn*	periostin, osteoblast specific factor	1423606_at	cell adhesion
			
**Protein targeting**			
*Arl6ip1*	ADP-ribosylation factor-like 6 interacting protein 1	1451131_at	cotranslational protein targeting to membrane
*Tmod1*	tropomodulin 1	1422754_at	myofibril assembly; muscle thick filament assembly
			
**Transcription, processing, and chromatin structure**			
*Hp1 bp3*	heterochromatin protein 1, binding protein 3	1415751_at	nucleosome assembly
*Klf15*	Kruppel-like factor 15	1448181_at	regulation of transcription, DNA-dependent
*Rora*^†^	RAR-related orphan receptor alpha	1420583_a_at	regulation of transcription, DNA-dependent
*Slu7*	SLU7 splicing factor homolog	1420030_at	alternative nuclear mRNA splicing, via spliceosome

* These genes were identified after bioinformatic analysis filtering first on biological patterns using both Genespring and Spotfire Software followed by filtering on GO biological categories using the DAVID Bioinformatics Database. ^†^These genes were down-regulated ≥ 2-fold in BALB^*Lsp*-*d *^vs. BALB 1 day following protocol 1 (chronic BHT) (P < 0.05)

An additional expression profile identified by unsupervised analysis only consists of genes with decreased transcript levels 1 day following BHT treatment that recover to control levels in BALB mice 3 days following BHT, but are still depressed in BALB^*Lps*-*d *^(see Additional file 2, Table S1, Tab A, column N).

**Figure 2:**
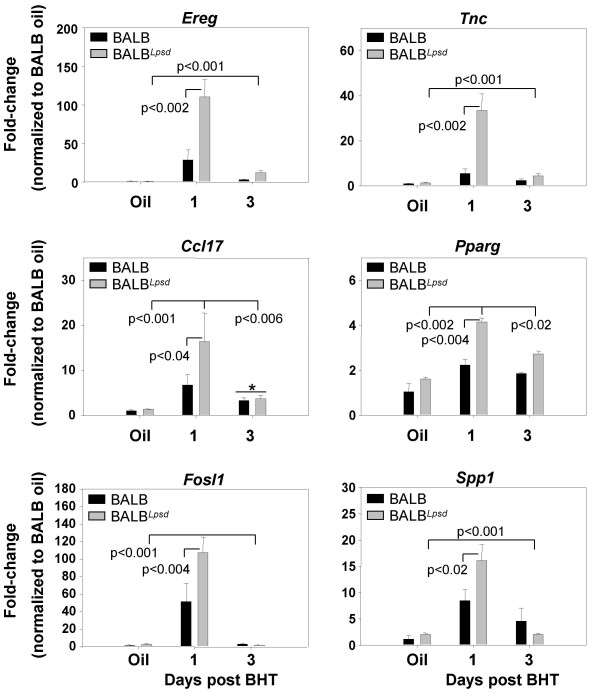
**Confirmation of select genes identified using protocol 1 (chronic BHT) in BALB and BALB^*Lps*-*d *^mice**. These genes were selected using the supervised method of microarray analysis in both strains 1 and 3 days following BHT. Mean and SEM are presented; n = 3 mice/experimental group for all. P values are indicated on the graphs for each gene; the lines indicate comparisons between BHT treatment and corn oil control vehicle or between BALB^*Lps*-*d *^compared to BALB mice. * P < 0.05 for 3 day following BHT treatment versus corn oil vehicle for Ccl17, and no difference was found between BALB^*Lps*-*d *^and BALB mice.

We confirmed the change in transcript level seen in the microarray analysis using qRTPCR. We used RNA from the same samples used for microarray analysis and, in the case of Protocol 2, samples from a repeat study with the same design. qRTPCR analyses confirmed the changes in expression seen by microarray analysis in genes listed in Table 1 (epiregulin (*Ereg*), tenacin C (*Tnc*), secreted phosphoprotein 1 (*Spp1*), and peroxisome proliferator activated receptor gamma (*Pparg*)), as well as other genes with profile "BHT-1day_up" (chemokine (C-C motif) ligand 17 (*Ccl17*), fos-like antigen 1 (*Fosl1*)) that did not fall below the p value for DAVID, but were significantly different between strains (Figure 2).

### Pulmonary inflammation differences between the BALB vs. BALB^*Lps*-*d *^mice during the progression stage of tumorigenesis (Protocol 2, "Progression")

**Figure 3:**
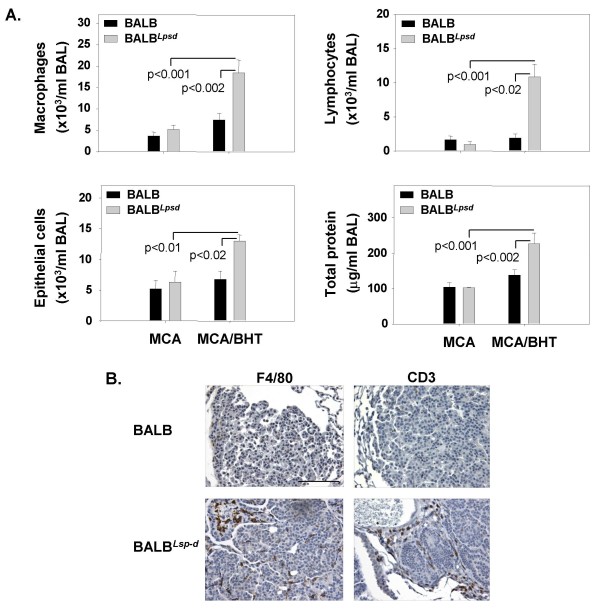
**Comparison of inflammatory infiltrates in tumor-bearing BALB and BALB^*Lps*-*d *^mice 27 wks post carcinogen**. A. Total BALF macrophages, lymphocytes, epithelial cells and protein recovered from BALB and BALB^*Lps*-*d *^mice after MCA or MCA/BHT treatment. Significant increases in inflammation in tumor-bearing mice were found in BALB^*Lps*-*d *^compared to BALB mice. Mean and SEM are presented; n = 6 mice/experimental group. P values are indicated on the graphs for each phenotype (macrophages, lymphocytes, epithelial cells, and total protein); comparisons are between MCA/BHT treatment groups and MCA/oil vehicle or between BALB and BALB^*Lps*-*d *^mice. B. Immunohistological staining in BALB and BALB^*Lps*-*d *^mice after 27 wks after MCA/BHT treatment for macrophages and T-lymphocytes using F4/80 and CD3 markers, respectively. Bar indicates 100 μm. Slides were counterstained with hematoxylin.

BALB^*Lpsd *^mice develop significantly more tumors after MCA/BHT than wild type BALB mice or than BALB^*Lpsd *^mice after MCA/oil treatment [20]. We found mean BALF total protein content and numbers of macrophages, lymphocytes, and epithelial cells were significantly increased during progression (p < 0.05) in the tumor bearing MCA/BHT BALB^*Lps*-*d *^mice compared to the BALB^*Lps*-*d *^MCA/oil controls (Figure 3A). No increases in inflammatory cell infiltrates were found in the wildtype BALB mice after either MCA/oil or MCA/BHT.

Histological staining of the same lungs with anti-F4/80 or anti-CD3 markers for macrophages or T-lymphocytes, respectively, confirmed the BALF results (Figure 3B). Anti-F4/80 and anti-CD3 were found primarily adjacent to but not in the tumor parenchyma in BALB^*Lps*-*d *^mice; this peritumoral staining was not seen in BALB mouse tumors.

Adenoma tumor size and morphology were not significantly different between the strains (data not shown), suggesting the difference in inflammation is not due to different stages of tumorigenesis or different tumor morphologies. Few F4/80- or CD3-positive-staining macrophages or lymphocytes were found in the MCA/oil lung sections from either strain (data not shown).

### Transcriptome changes between the BALB vs. BALB^*Lps*-*d *^mice during the progression stage of tumorigenesis (Protocol 2, "Progression")

**Figure 4:**
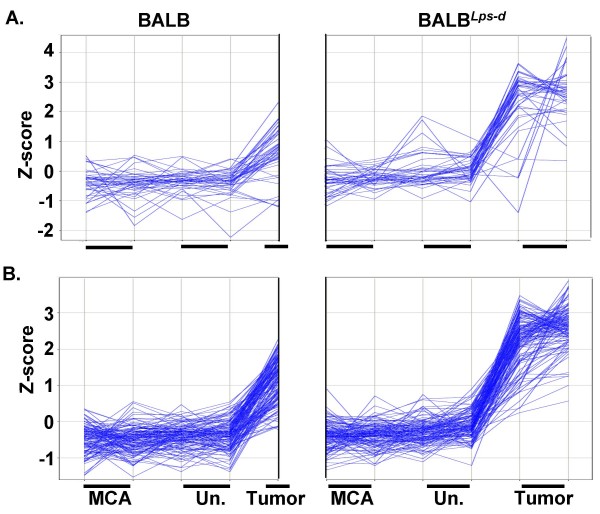
**Analysis of protocol 2 genes increased in the BALB^Lps-d ^tumors compared to BALB tumors**. Supervised and unsupervised bioinformatic methods were used for these analyses. A. Cluster 4 identified using statistical (Genespring) analysis. Each vertical line represents one sample for each strain for an n = 2 (Un = uninvolved, MCA = 3-methlycholanthrene, and for BALB^*Lps*-*d *^tumor) with the exception of BALB tumor (n = 1), indicated by the small horizontal black line above each treatment on the x-axis. B. Profile (Spotfire) analysis similar to that of cluster 4. Z-score (y-axis) is the intensity in terms of standard deviations (SD) from the mean. The z-score is calculated by subtracting the mean and dividing by the SD for each probeset. See Additional file 6, Figure S3 for supervised and unsupervised analysis depicted as heat maps.

We next assessed gene expression differences in control (MCA/oil) tissue, MCA/BHT dissected tumor tissue, and MCA/BHT uninvolved tissue, i.e. tissue remaining after tumor dissection in the same mice. We identified three biological profiles: up in tumor tissue relative to MCA alone or uninvolved tissue in the BALB mice and compared to the tumor tissue in BALB^*Lps*-*d *^mice ("up in WT tumor"); up in tumor tissue compared to MCA alone or uninvolved tissue, and more so in BALB^*Lps*-*d *^than BALB ("up in KO tumor"; Figure 4 and Additional file 6, Figure S3); and down in tumor compared to MCA alone or uninvolved tissue, and more so in BALB than in BALB^*Lps*-*d *^("down in WT tumor"). As before, we found that supervised and unsupervised analysis methods identified overlapping response profiles, which we combined into the three profiles described (i.e. 6 gene expression patterns, 3 per analysis). All genes in these profiles are listed in Additional file 2, Table S1, Tab A. We then analyzed GO pathways for the genes in these three profiles using DAVID (Additional file 2: Table S1, Tab B, genes within selection criteria; Tab D are the GO categories for late genes). Transcripts with "up in KO tumor" profiles are associated with immune response, inflammatory response and chemotaxis, apoptosis and cell death, protein synthesis and turnover, and cell cycle and cell growth (Table 3). Transcripts with "down in WT tumor" profiles are primarily associated with immune response and inflammation (Table 4). Transcripts with "up in WT tumor" profiles are associated with angiogenesis, protein synthesis, and immune responses.

**Table 3:** Representative gene expression changes increased in BALB^Lps-d^ tumors.


**Gene Symbol**	**Gene Name**	**Affymetrix Probe ID**	**Major GO Category(ies)**

**Angiogenesis**			
*Anpep*	alanyl aminopeptidase	1421424_a_at	angiogenesis; proteolysis
*Col18a1*^†^	procollagen, type XVIII, alpha 1	1418237_s_at	angiogenesis
*Nus1*	nuclear undecaprenyl pyrophosphate synthase 1 homolog	1419915_at	angiogenesis; multicellular organismal development
			
**Apoptosis and cell death**			
*Bcl2l14*	bcl2-like 14	1424814_a_at	apoptosis
*Cideb*^†^	cell death-inducing DNA fragmentation factor, alpha subunit-like effector B	1418976_s_at	apoptosis; induction of apoptosis
*Pglyrp1*	peptidoglycan recognition protein 1	1449184_at	apoptosis; immune response
			
**Cell cycle and cell growth**			
*Aspm*	asp-like, microcephaly associated	1422814_at	cell cycle; mitosis
*Ereg*^†^	Epiregulin	1419431_at	regulation of progression through cell cycle; angiogenesis
*Cenpf*	centromere protein F	1427161_at	G2 and M phase of mitotic cell cycle
*Mycn*	v-myc myelocytomatosis viral related oncogene, neuroblastoma derived		regulation of progression through cell cycle; regulation of transcription, DNA-dependent
			
**Immune response**			
*Clcf1*	cardiotrophin-like cytokine factor 1	1437270_a_at	cell surface receptor linked signal transduction; JAK-STAT cascade
*Flt3l*	FMS-like tyrosine kinase 3 ligand	1422115_a_at	lymphocyte differentiation
*H2-oa*	histocompatibility 2, O region alpha locus	1419297_at	immune response; antigen processing and presentation
*Klrb1b*	killer cell lectin-like receptor subfamily B member 1B	1420421_s_at	negative regulation of natural killer cell mediated cytotoxicity
*Raet1a*	retinoic acid early transcript 1, alpha	1420603_s_at	positive regulation of immune response to tumor cell
			
**Inflammatory response and chemotaxis**			
*Ccr*^1^	chemokine (C-C motif) receptor 1	1419610_at	inflammatory response; signal transduction
*Cxcl4*^†^; *Pf4*	platelet factor 4; chemokine (C-X-C motif) ligand 4	1448995_at	chemotaxis; immune response
*Cxcl5, Ena-* 78	chemokine (C-X-C motif) ligand 5	1419728_at	chemotaxis; inflammatory response
*Cxcl9*^†^, *Mig*	chemokine (C-X-C motif) ligand 9	1418652_at	inflammatory response; immune response
*Cxcl11*^†^, *Itac*	chemokine (C-X-C motif) ligand 11	1419697_at	chemotaxis; inflammatory response
*Kng1*	kininogen 1	1416676_at	inflammatory response; blood coagulation
*Spp1*^†^	secreted phosphoprotein 1	1449254_at	ossification; anti-apoptosis
			
**Protein synthesis and turnover**			
*Cdkn2a*^†^	cyclin-dependent kinase inhibitor 2A	1450140_a_at	regulation of cyclin-dependent protein kinase activity; cell cycle
*Cfi*^†‡^	Complement component factor i	1418724_at	proteolysis; immune response
*Gzme*	granzyme E	1421227_at	proteolysis; cytolysis
*F*10	coagulation factor X	1449305_at	proteolysis

These genes were identified in the "Up-in-KO_tumor" profile (A) and "Down in WT tumor" profile (B) that are below the p value set for the DAVID analysis (p < 0.0001); see Additional file 2, Table S1 for the complete profile gene lists.

*Representative increased genes from the "Up-in-KO_tumor" profile that were higher in the BALB^*Lpsd *^tumors compared to BALB mice. These genes were identified after bioinformatic analysis filtering first on biological patterns using both Genespring and Spotfire Software followed by filtering on GO biological categories using the DAVID Bioinformatics Database. ^†^Genes upregulated ≥ 1.5-2-fold in BALB^*Lspd *^tumors vs. BALB tumors based on GeneSpring. ^‡^Genes with significant interaction effects comparing strain (BALB vs BALB^*Lspd*^) and treatment (MCA, MCA/BHT tumors, MCA/BHT uninvolved tissue) (p < 0.05). Bolded genes are those genes in common with the gene list identified for the 4BHT study (see Table 1).

**Table 4:** Representative gene expression changes decreased in BALB tumors.

Gene Symbol	Gene Name	Affymetrix Probe ID	Major GO Category(ies)
**Angiogenesis**			
*F*13*a*1	coagulation factor XIII, A1 subunit	1448929_at	tRNA aminoacylation for protein translation; blood coagulation
*Kdr*^†^	kinase insert domain protein receptor	1449379_at	angiogenesis; ovarian follicle development
*Tbx1*^†^	T-box 1	1425779_a_at	angiogenesis; blood vessel development
			
**Apoptosis and cell death**			
*Rassf5*	ras association (RalGDS/AF-6) domain family 5	1422637_at	apoptosis; cell cycle
*Unc5c*	unc-5 homolog C	1449522_at	apoptosis; signal transduction
			
**Immune response**			
*Cd3d*^†^	CD3 antigen, delta polypeptide	1422828_at	protein complex assembly; cell surface receptor linked signal transduction
*Cd79a*	CD79A antigen (immunoglobulin-associated alpha)	1418830_at	immune response; cell surface receptor linked signal transduction
*Cr2*^†^	complement receptor 2	1425289_a_at	immune response; complement activation, classical pathway
*Ltb*	lymphotoxin B	1419135_at	immune response; lymph node development
*Ppbp*^†^, *Cxcl7*	pro-platelet basic protein	1418480_at	immune response
*Slamf1*	signaling lymphocytic activation molecule family member 1	1425570_at	lymphocyte activation
*Tnfrsf13c*	tumor necrosis factor receptor superfamily, member 13c	1419307_at	B cell homeostasis; positive regulation of germinal center formation
			
**Inflammatory response and chemotaxis**			
*Ccl5*^†^	chemokine (C-C motif) ligand 5	1418126_at	chemotaxis; inflammatory response
*Ccr2*	chemokine (C-C motif) receptor 2	1421186_at	chemotaxis; inflammatory response
*Chst1*	carbohydrate (keratan sulfate Gal-6) sulfotransferase 1	1449147_at	carbohydrate and galactose metabolic process
*Enpp2*	ectonucleotide pyrophosphatase/phosphodiesterase 2	1415894_at	chemotaxis; metabolic process
*S*100*a*8	S100 calcium binding protein A8	1419394_s_at	chemotaxis
			
**Protein synthesis and turnover**			
*Cfd*	complement factor D (adipsin)	1417867_at	proteolysis; immune response
*Gzma*	granzyme A	1417898_a_at	proteolysis; apoptosis
			
**Transcription, processing, and chromatin structure**			
*Hipk2*	homeodomain interacting protein kinase 2	1429566_a_at	negative regulation of transcription from RNA polymerase II promoter
*Myst3*	MYST histone acetyltransferase 3	1436315_at	nucleosome assembly
*SpiB*	Spi-B transcription factor	1460407_at	regulation of transcription, DNA-dependent

These genes were identified in the "Up-in-KO_tumor" profile (A) and "Down in WT tumor" profile (B) that are below the p value set for the DAVID analysis (p < 0.0001); see Additional file 2, Table S1 for the complete profile gene lists.

*Representative decreased genes from the "Down in WT tumor" profile for genes BALB tumors compared to BALB^*Lsp*-*d *^tumors. These genes were identified after bioinformatic analysis filtering first on biological patterns using both Genespring and Spotfire Software followed by filtering on GO biological categories using the DAVID Bioinformatics Database. ^†^Genes down-regulated ≥ 50% in BALB tumors compared to BALB^*Lspd *^tumors based on GeneSpring.

**Figure 5:**
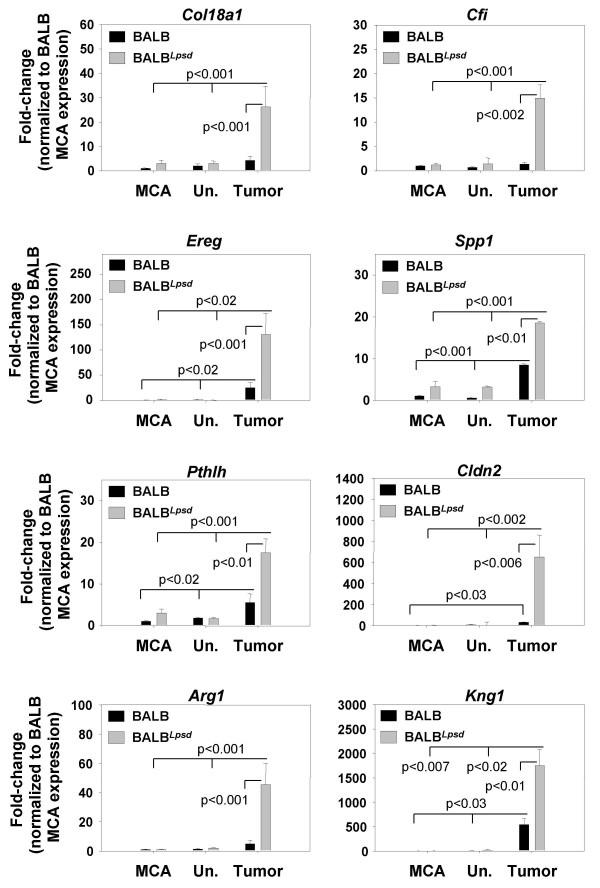
**Confirmation of select genes identified during progression (Protocol 2; MCA/BHT) in the BALB and BALB^*Lps*-*d *^mice**. These genes were selected based on the supervised microarray analysis in both strains 27 wks following MCA/BHT. Genes upregulated in the BALB^*Lps*-*d*^mice compared to BALB mice. Mean and SEM are presented; n = 4-5 mice/experimental group. P values are indicated on the graphs for each gene; comparisons are between MCA/BHT treatment and MCA/oil controls or between BALB^*Lps*-*d *^compared to BALB mice.

qRTPCR was done to confirm the expression identified by microarray analysis (Figure 5) on select genes from Table 3 (collagen, type XVIII, alpha 1 (*Col18a1*), complement component factor i (*Cfi*), *Ereg*, kininogen 1 (*Kng1*), *Spp1*) that varied between the tumors in BALB and BALB^*Lps*-*d *^mice, and three additional genes (parathyroid hormone-like peptide (*Pthlh*), claudin 2 (*Cldn2*), arginase 1 (*Arg1*)) that were highly, significantly upregulated in the BALB^*Lpsd *^tumors compared to BALB tumors, but did not fall below the p value set for the DAVID analysis. Two genes in Table 1 from the early protocol (*Ereg *and *Spp1) *were also identified with Protocol 2 (late, progression stage) possibly indicating transcripts that are involved in both promotion and progression of tumorigenesis.

**Figure 6:**
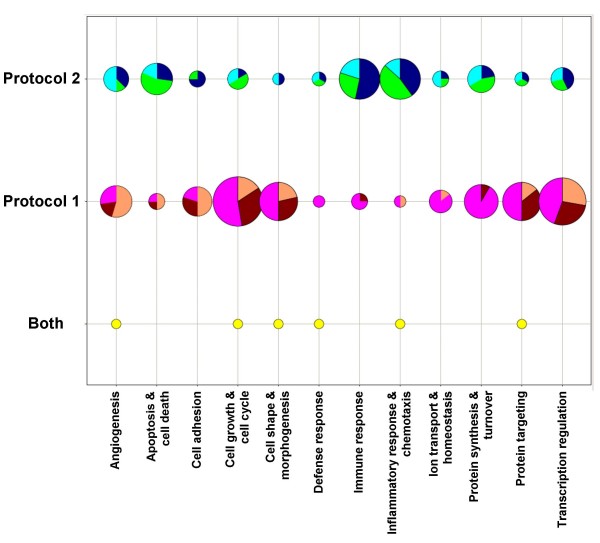
**Pie chart comparison of the genes involved during early and late stages of carcinogenesis**. Protocol 1 (promotion; Figure 1A-B) and protocol 2 (advanced; Figure 4A-B) genes representing the major GO categories for these early and more advanced events during tumorigenesis are compared. Pie charts represent the number of genes in each group; largest circle contains 19 genes, the smallest 1. Protocol 2 colors: Down_BALB_Tumor, navy; Up_BALB_Tumor, aqua; Up_BALB^*Lps*-*d*^_Tumor, bright green. Protocol 1 colors: Down_BALB^*Lps*-*d*^_BHT_1dy, maroon; Up_BALB^*Lps*-*d *^_BHT_1dy, magenta; BHT_1day_BALB_recovery, orange. Genes selected in both Protocols are in yellow.

We then compared the summary GO categories for the genes identified using Protocol 1 (tumor promotion; early) and Protocol 2 (tumor progression; late). Pie charts illustrate the number of genes in each of 12 summary categories with altered transcript levels at early and advanced stages of carcinogenesis (Figure 6). This figure demonstrates an increase in the number of genes in cell growth, shape and protein synthesis and targeting, and transcription categories early in the response followed by a switch to more inflammation and immune responses, as well as apoptosis, protein synthesis, and cell growth, during the advanced stages of tumorigenesis.

To better understand the role of the microenvironment in the uninvolved tissue from tumor-bearing lungs in BALB and BALB^*Lpsd *^mice, we then analyzed differential transcript expression in uninvolved tissue from tumor bearing lungs. Additional file 2, Table S1 contains those genes differentially expressed in uninvolved tissue between strains (all have p < 0.05 and two-fold or greater change in expression). The most dominant GO categories in BALB^*Lps*-*d *^uninvolved tissue were immune response (*p *< 0.001) and immune system process (*p *< 0.005) with those genes upregulated ≥ 2 fold in BALB^*Lps*-*d *^uninvolved tissue compared to BALB uninvolved tissue (e.g. chemokine (C-C motif) receptor 1 (*Ccr2*; p < 0.05) (Additional file 2: Table S1, Tab A and Tab F). The major categories in BALB uninvolved tissue were hemopoietic cell lineage (*p *< 0.004), immune system process (*p *< 0.015), leukocyte differentiation (*p *< 0.027) for those genes 50% down-regulated (e.g. chemokine (C-C motif) ligand 5 (*Ccl5*), CD3 antigen, delta polypeptide (*Cd3d*)) compared to BALB^*Lps*-*d *^(Additional file 2: Table S1, Tab A and Tab E). The analysis of uninvolved tissue supports the above transcript analysis in micro-dissected tumors where more immune and inflammatory transcripts were elevated in BALB^*Lpsd *^and decreased in BALB mice.

## Discussion

We previously demonstrated that lung carcinogenesis was enhanced in mice with dysfunctional *Tlr4*, likely due to greater chronic inflammation during tumor promotion in the *Tlr4 *mutant mice relative to the *Tlr4 *wild type strain [20]. In the present study, we found that TLR4 also protects against the inflammation observed during the advanced stages of tumorigenesis (see Figure 3) which supports our previous findings [20]. Using transcriptomics and integrative biology, we have also identified *Tlr4*-modulated gene expression pathways that distinguish the tumor promotion stage (Protocol 1) and the progression stage (Protocol 2) of tumorigenesis. These novel *Tlr4*-mediated pathways and gene signatures provide insight to the protective effect of TLR4 against lung carcinogenesis and may provide a means to develop diagnostic tests to identify individuals at risk for the disease.

We identified many novel genes with increased transcript levels in *Tlr4*-mutated mice (BALB^*Lps*-*d*^) after BHT. These include *Ereg*, heparin-binding EGF-like growth factor (*Hbegf*), high mobility group AT-hook 1 (*Hmga1*), myelocytomatosis oncogene (*Myc*), *Spp1*, and *Tnc*, and they are associated with GO categories that are consistent with early stages of carcinogenesis (cell cycle and growth, angiogenesis, protein synthesis and targeting, and transcription). *Ereg *and *Hbegf *are epidermal growth factor receptor (EGFR) ligands that can bind and activate the ERBB family of receptors [27]. EREG is an intratumoral marker for advanced NCSLC [28] and is upregulated in cancer cell lines [29,30]. *Spp1 *is a chemokine-like protein that appears to have a role in macrophage infiltration, among other functions [31], and has been identified as a lung AC biomarker [32]. *Spp1 *can also be activated by Kirstein ras proto-oncogene (K-RAS) in lung neoplasia [33]. Because *Ereg *and *Spp1 *(among others) are elevated at early (during promotion) and late (during progression) stages, these genes may play a role in the transition from initiated cell through promotion (adenomas) to progression (carcinomas).

Mutated *Tlr4 *caused increased inflammation in tumor-bearing mice (Figure 3). The majority of the transcripts that were significantly decreased in the uninvolved tissue in the BALB mice compared to BALB^*Lps*-*d *^mice were from genes involved in inflammation and immune responses, e.g. *Ccl5 *and *Cd3d *(Additional file 2, Table S1). Additionally, transcripts from immune response genes (such as *Ccr2) *were increased in the BALB^*Lps*-*d *^uninvolved tissue compared to BALB uninvolved tissue. Thus, these data are consistent with the increased inflammation observed in the BALF from BALB^*Lps*-*d *^mice (Figure 3). For example, CCR2 is the receptor for monocyte chemoattractant protein-1 (MCP-1) that modulates the innate immune response and recruits monocytes/macrophages to sites of inflammation [34]. In uninvolved tissue from the BALB^*Lps*-*d *^mice treated with MCA/BHT, we found a 1.5-fold increase in MCP-1 protein levels above MCA/oil controls compared to no increase in the BALB mice (preliminary ELISA analysis not shown). Because macrophages were recruited to the lungs but do not infiltrate the tumors [35], it is not surprising that chemokines specific to these cell types were upregulated in uninvolved tissue.

Some overlap in biological functions were found based on our analysis (see Fig 6). For example, GO categories for cell cycle and growth (*Ereg*), inflammation (*Spp1*), and development (ornithine decarboxylase (*Odc1*)) contained genes identified in promotion and progression expression patterns for BALB^*Lpsd *^upregulated genes. *Arg1 *induces polyamine synthesis and hence proliferation, through ODC1 and was also significantly up-regulated in the BALB^*Lps*-*d *^tumors (Figure 5; [35]).

**Figure 7:**
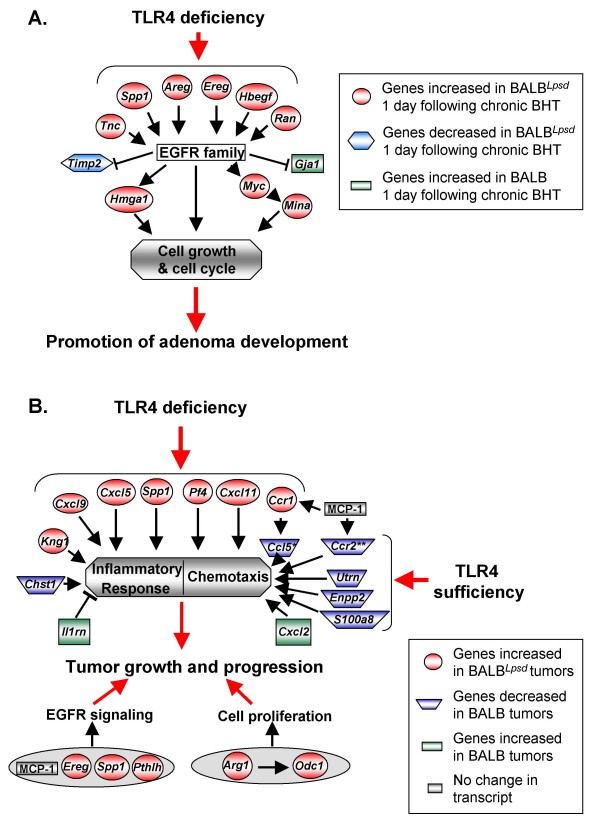
**Proposed pathways for promotion (A) and progression genes (B) influenced by TLR4**. A. Promotion genes primarily signal through or are involved in EGFR signaling which may lead to cell growth and eventual tumor development. Thus, the EGFR pathway may drive the proliferative response in mice deficient in *Tlr4 *and concurrently inhibit communication between cells (*Gja1*), thus providing a growth advantage. References for gene interactions not described in text: *Ran*, [49]; *Myc*, [50]; *Hmag1*, [51]; *Tnc*, [52]; *Mina*, [53], *Timp2 *[54]. Symbols: red, genes increased in BALB^*Lps*-*d *^mice 1 day Protocol 1; blue, genes decreased in BALB^*Lps*-*d *^mice 1 day Protocol; green, genes increased in BALB mice 1 day Protocol 1. B. During progression, the combination of inflammation observed in BALB^*Lps*-*d *^mice in concert with up-regulation of these and other immune response genes, the many inflammation and immune response genes that were down regulated in BALB mice, and the additional contribution of cell growth genes (e.g. SPP1; [31], MCP-1; [55], supports a role of TLR4 in protection against lung tumor progression. Symbols: red, genes increased in BALB^*Lps*-*d*^tumors; blue, genes decreased in BALB tumors; green, genes increased in BALB tumors; grey, protein changes in uninvolved tissue from the BALB^*Lps*-*d *^mice. *Also decreased in BALB uninvolved tissue; **Also increased in BALB^*Lps*-*d *^uninvolved tissue. Abbreviations: *Ccr2*, chemokine (C-C motif) receptor 1; *Chst1*, carbohydrate sulfotransferase 1, *Cxcl2*, chemokine (C-X-C motif) ligand 2; *Enpp2*, ectonucleotide phosphodiesterase 2; *Il1rn*, interleukin 1 receptor antagonist; *S100a8*, S100 calcium binding protein A8; *Timp2*, tissue inhibitor of metalloproteinases; *Utrn*, utrophin.

Based on the major GO categories identified, we propose that several key pathways are influenced by TLR4 deficiency during carcinogenesis (Figure 7). Many of the genes identified during promotion (e.g. *Ereg*, *Hbegf*, *and Spp1) *can signal through EGFR or are involved in EGFR signaling events which can then lead to cell growth and eventual tumor development (Figure 7A). Other genes inhibited by EGFR signaling, such as gap junction protein alpha 1 (*Gja1*), a mediator for cell-cell communication in lung epithelial cells that is down-regulated in human and mouse lung cancer [36,37], were elevated in the BALB mice (*Gja1*, confirmed by PCR, data not shown). Thus, during promotion, the EGFR pathway may drive the proliferative response in the *Tlr4 *mutant mice while inhibiting cell-cell communication, which provides a growth advantage for the initiated cells. During progression (Figure 7B), many inflammation, chemotactic and immune response genes were elevated in the BALB^*Lps*-*d *^mice and decreased in the BALB mice, likely providing a more aggressive microenvironment for tumor growth. At the same time, several proliferative pathways were elevated in the BALB^*Lps*-*d *^mice (e.g. EGFR), further increasing the chance of tumor growth and progression. Thus, based on our analysis of pathways downstream of TLR4 or those downstream of mice lacking TLR4, it will be important to focus future studies on these specific pathways to further elucidate the mechanisms of TLR4-mediated protection against BHT-induced inflammation and tumor promotion, as the two phenomena are certainly linked. However, other genes that were over the set p value for the DAVID analysis with the progression study (and thus not found in Tables 3 and 4) but that were identified in the "up_KO_tumor" profile, provide additional evidence for similarities between the early and advanced stages of carcinogenesis. For example, *Pthlh *(Figure 5) is located in the pulmonary adenoma susceptibility 1 (*Pas1*) gene cluster near *Kras *on mouse chromosome 6 [38]. *Pthlh *is also associated with decreased survival in lung AC patients [39] possibly through EGFR ligand activation [33]. In addition, PTHLH induces MCP-1 in prostate cancer which can lead to increased proliferation [40].

Several investigations have characterized gene transcripts in human and mouse lung AC [25,41-45]. Stearman et al. [25] compared human and mouse genes responsible for lung AC; one gene identified was (*Spp1*) that was increased in BALB^*Lps*-*d *^during both protocols (Tables 1, 2, 3 and 4). However, several other genes in our study were either significantly upregulated (such as *Tyms*, thymidylate synthase) or downregulated (such as *Aldh1a1*, aldehyde dehydrogenase 1a1, and *Vwf*, Von Willebrand factor) in the tumors from both strains (all ≥ 2-fold or ≥ 50%; p < 0.05), similar to the A/J mice used by Stearman et al. These genes may be more general markers of advanced stages of tumorigenesis and their identification in our study supports the comparison between species and strain. The mouse lung AC model was similar to human AC in the molecular changes that occurred during carcinogenesis [25]. In a microarray study using *K-ras *over-expressing mice, *Ereg *was identified as a significantly upregulated transcript in lung tumors [43], which also supports our results. Additional human microarray studies that assessed lung tissue from smokers [44], and tumor tissue from patients with AC and squamous cell carcinoma, another NSCLC, identified other unique genes [41,42,45] such as *Cdkn1a*, cyclin-dependent kinase inhibitor 1A (P21), up in BALB^*Lps*-*d *^(Table 1). Thus, a number of the genes identified in the present study were also identified in human studies and were all associated with either inflammation or cell growth. However, due to the specific TLR4 focus in our model, as well as differences in tumor histology and grade, tumor size, node status, and margin status, many of the genes we identified were not found in these human and mouse microarray studies. In addition, the numbers of the presumptive bronchioalveolar stem cells (BASCs) expressing both a type II cell (prosurfactant apoprotein C; SPC) and Clara cell specific marker (Clara cell 10 kD protein; CC10) may also influence gene expression in these different models [46].

## Conclusion

In summary, based on our transcriptome analysis, the protective effect of TLR4 in lung carcinogenesis involves inhibition of multiple pathways, many of which overlap and likely interact, such as the EGFR and PTHLH pathways (Figure 7). EGFR activating mutations in human NSCLC correlates to positive Gefitinib (EGFR kinase inhibitor) responsiveness, thus deficiency in TLR4 in humans may further activate this pathway [47,48]. These studies have identified a novel panel of genes differentially expressed in mice with sufficient or deficient TLR4 during tumor promotion (such as those genes up-regulated in BALB^*Lps*-*d*^: amphiregulin (*Areg*), *Ereg*, *Hbegf*, *Hmga1*, *Myc*, myc induced nuclear antigen (*Mina*), ras-related nuclear protein (*Ran*), *Spp1*, and *Tnc; *see Figure 7A) and during progression stages of tumorigenesis (such as genes up-regulated in BALB^*Lps*-*d *^tumors: *Arg1*, chemokine (C-C motif) receptor 1 (*Ccr1*), platelet factor 4 (*Pf4*), chemokine (C-X-C motif) ligand 5 (*Cxcl5*), *Cxcl9, Cxcl11, Ereg, Kng1, Odc1*, *Pthlh*, and *Spp1*, see Figure 7B). These gene panels for early and advanced stages of tumorigenesis may be predictive determinants for tumor susceptibility in those individuals with altered innate immune systems. The mouse model will allow us to test the significance of these genes in lung AC using *in vivo *and *in vitro *molecular techniques, such as transgenic or knock-out mice/cells, as well as siRNA, in order to elucidate additional pathways to target for future therapies. In addition, these gene profiles may aid in our understanding of those individuals occupationally exposed to endotoxin who appear to have reduced lung cancer risk [15-17].

## Competing interests

The authors declare that they have no competing interests.

## Authors' contributions

AKB designed and performed the mouse experiments including collecting and analyzing data, RNA isolation, Genespring analysis, PCR, and wrote the manuscript. J. Fostel performed the unsupervised microarray analysis with Spotfire and assisted in writing the manuscript. LMD and CW assisted in the mouse experiments. EAR performed data analysis. SFG performed the microarray analyses, from RNA purity testing to hybridization and array scanning. JF assisted in the immunohistochemical staining. SRK assisted in the microarray bioinformatic analysis and the writing of the manuscript. All authors read and approved the final manuscript.

## Supplementary Material

Additional file 1 **Additional Figure 1.** Experimental design. A) Protocol 1 (Promotion stage) involved exposure to BHT (150-200 mg/kg/week) or oil control in four weekly ip injections. Mice were sacrificed 1 and 3 days following the last dose of BHT and processed for BAL analysis and RNA extraction. B) Protocol 2 (Progression stage) involved a single injection of the initiator MCA (10 μg/g) followed by 6 weekly ip injections of either BHT (125-200 mg/kg/week; "promoter") or oil. Mice were sacrificed 27 weeks following the MCA exposure. Tumors and adjacent uninvolved tissue were micro-dissected for transcriptomic analysis, as well as assessment of pulmonary inflammation by BAL analysis and histology. Click here for file

Additional file 2 **Additional Table 1: A comprehensive compilation of all the data used for the analyses**. A) All microarray probes (Tab A) and profile(s) to which they were assigned. Flag columns: early clusters (columns D, E, L, M, N); late clusters (columns F, G, H, I, J, K). Data columns: BHT_1day up_KO_supervised (L) = early pattern (cluster 3), genes upregulated early in BALB^*Lpsd *^1 day following BHT vs. BALB; BHT_1day up_KO_unsupervised (E), genes with similar patterns as cluster 3 early (L); BHT_1day down_KO_supervised (M) = cluster 4 early, genes downregulated early (1 day following BHT) in BALB^*Lpsd *^vs. BALB; BHT_1day_down_KO_unsupervised (D), patterns similar to cluster 4 early (M); BHT_WT_recovery_unsupervised (N) genes with late recovery BALB^*Lpsd*^; Up_WT_tumor_supervised (F) = cluster 1 late, genes upregulated in BALB tumors vs. BALB^*Lpsd*^; Up_WT_tumor_unsupervised (J), patterns similar to cluster 1 late (F); Up_KO_tumor_supervised (G) = cluster 4 late, genes up-regulated in BALB^*Lpsd *^tumors vs. BALB; Up_KO_tumor_unsupervised (H), patterns similar to cluster 4 late (G); Down_WT_tumor-supervised (K) = cluster 5 late, genes down-regulated in BALB tumors vs. BALB^*Lpsd*^; Down_WT_tumor_unsupervised (I), patterns similar to cluster 5 late (K). Uninvolved genes: increased or decreased in BALB uninvolved tissue vs. BALB^*Lpsd *^(B); increased or decreased in BALB^*Lpsd *^uninvolved vs. BALB (C). B) DAVID analysis genes (Tab B) enrichment criteria: *p *values below 3 × 10^-4 ^(protocol 1, early genes) and 1 × 10^-4 ^(protocol 2, late genes); non-specific categories (> 100 genes) not included. Categories based on primary and secondary biological significance (D and E, respectively). C. DAVID analysis (Tab C), protocol 1 early genes based on combination in columns D, E, L, M, and N (Tab A). Red font = GO categories for selected analysis (SM Table 1B); yellow = categories not included. D. DAVID analysis (Tab D), protocol 2 late genes based on combination in columns F, G, H, I, J, and K (Tab A). Color scheme as described in C. E. DAVID analysis, protocol 2 uninvolved BALB genes (Tab A, column B). F. DAVID analysis, protocol 2 uninvolved BALB^*Lpsd *^genes in A (Tab A, column C). Click here for file

Additional file 3 **Additional Methods for the Affymetrix Mouse 430A_MOE array analysis**. Detailed methodology for the microarray analysis studies. Click here for file

Additional file 4 **Additional Table 2**. Primer pairs used for the qRT-PCR analysis as described in methods, for Figs. 2 and 5. Click here for file

Additional file 5 **Additional Figure 2: Heat maps of the same representative gene lists identified for protocol 1 depicted in Figure **1. A) Expression pattern for cluster 3 (BHT_1day up_KO) identified from supervised analysis in chronic BHT-treated BALB and BALB^*Lpsd *^mice over a time course (oil, 1 and 3 days following BHT). Each column represents an individual animal. From left to right, columns 2,3,6 = BALB, oil; columns 1,4,5 = BALB, 3 dy BHT; columns 13-15 = BALB, 1 dy BHT; columns 7-9 = BALB^*Lpsd*^, oil; columns 10-12 = BALB^*Lpsd*^, 3 dy BHT; columns 16-18 = BALB^*Lpsd*^, 1 dy BHT. B) Unsupervised analysis resulting in similar expression patterns to that identified in (A). From left to right, columns 1-3 = BALB, oil; columns 4-6 = BALB^*Lpsd*^, oil; columns 7-9 = BALB, 3 dy BHT; columns 10-12 = BALB^*Lpsd*^, 3 dy BHT; columns 13-15 = BALB, 1 dy BHT; columns 16-18 = BALB^*Lpsd*^, 1 dy BHT. N = 3 per treatment group for each strain. Y-axis is the probeset. Click here for file

Additional file 6 **Additional Figure 3: Heat maps of the same representative gene lists identified from protocol 2 depicted in Figure **4. A) Expression pattern for cluster 4 (Up_KO_tumor) identified by supervised analysis. From left to right, columns 1-2 = BALB^*Lspd*^, MCA/BHT-induced tumor tissue; column 3 = BALB, MCA/BHT-induced tumor tissue; columns 4-5 = BALB^*Lspd*^, MCA/BHT-induced uninvolved tissue; columns 6-7 = BALB^*Lspd*^, MCA exposed tissue; columns 8, 11 = BALB, MCA exposed tissue; columns 9-10 = BALB, MCA/BHT-induced uninvolved tissue. B) Unsupervised analysis with similar gene expression patterns to that observed in (A). From left to right, column 1 = BALB, MCA/BHT-induced tumor tissue; columns 2-3 = BALB^*Lspd*^, MCA/BHT-induced tumor tissue; columns 4-5, BALB^*Lspd*^, MCA/BHT-induced uninvolved tissue; columns 6,10 = BALB^*Lspd*^, MCA exposed tissue; columns 7-8, BALB, MCA/BHT-induced uninvolved tissue; columns 10-11 = BALB, MCA exposed tissue. N = 2 per treatment group, except BALB tumor (n = 1). Y-axis is the probeset. Click here for file
