# Supplementary material for: Transcriptomic analysis of pathways regulated by toll-like receptor 4 in a murine model of chronic pulmonary inflammation and carcinogenesis
Source: Mol Cancer. 2009 Nov 19;8:107. doi: 10.1186/1476-4598-8-107 (PMC2785769; doi:10.1186/1476-4598-8-107)
Supplement: Additional file 1 — Additional Figure 1. Experimental design. A) Protocol 1 (Promotion stage) involved exposure to BHT (150-200 mg/kg/week) or oil control in four weekly ip injections. Mice were sacrificed 1 and 3 days following the last dose of BHT and processed for BAL analysis and RNA extraction. B) Protocol 2 (Progression stage) involved a single injection of the initiator MCA (10 μg/g) followed by 6 weekly ip injections of either BHT (125-200 mg/kg/week; "promoter") or oil. Mice were sacrificed 27 weeks following the MCA exposure. Tumors and adjacent uninvolved tissue were micro-dissected for transcriptomic analysis, as well as assessment of pulmonary inflammation by BAL analysis and histology. [file 1476-4598-8-107-S1.PPT]

## Slide 1
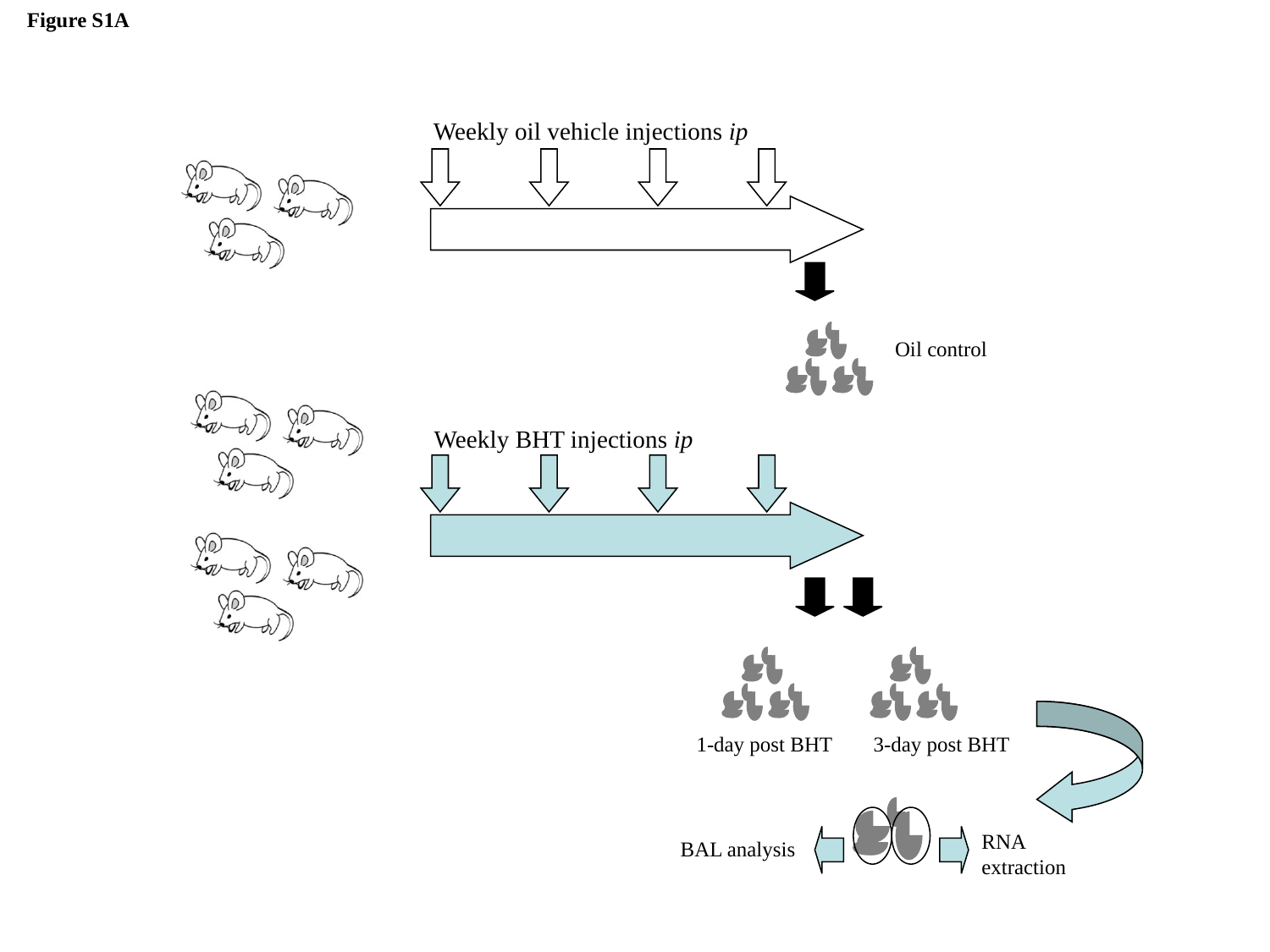

Figure S1A
Weekly oil vehicle injections ip
Oil control
Weekly BHT injections ip
1-day post BHT
3-day post BHT
BAL analysis
RNA extraction

## Slide 2
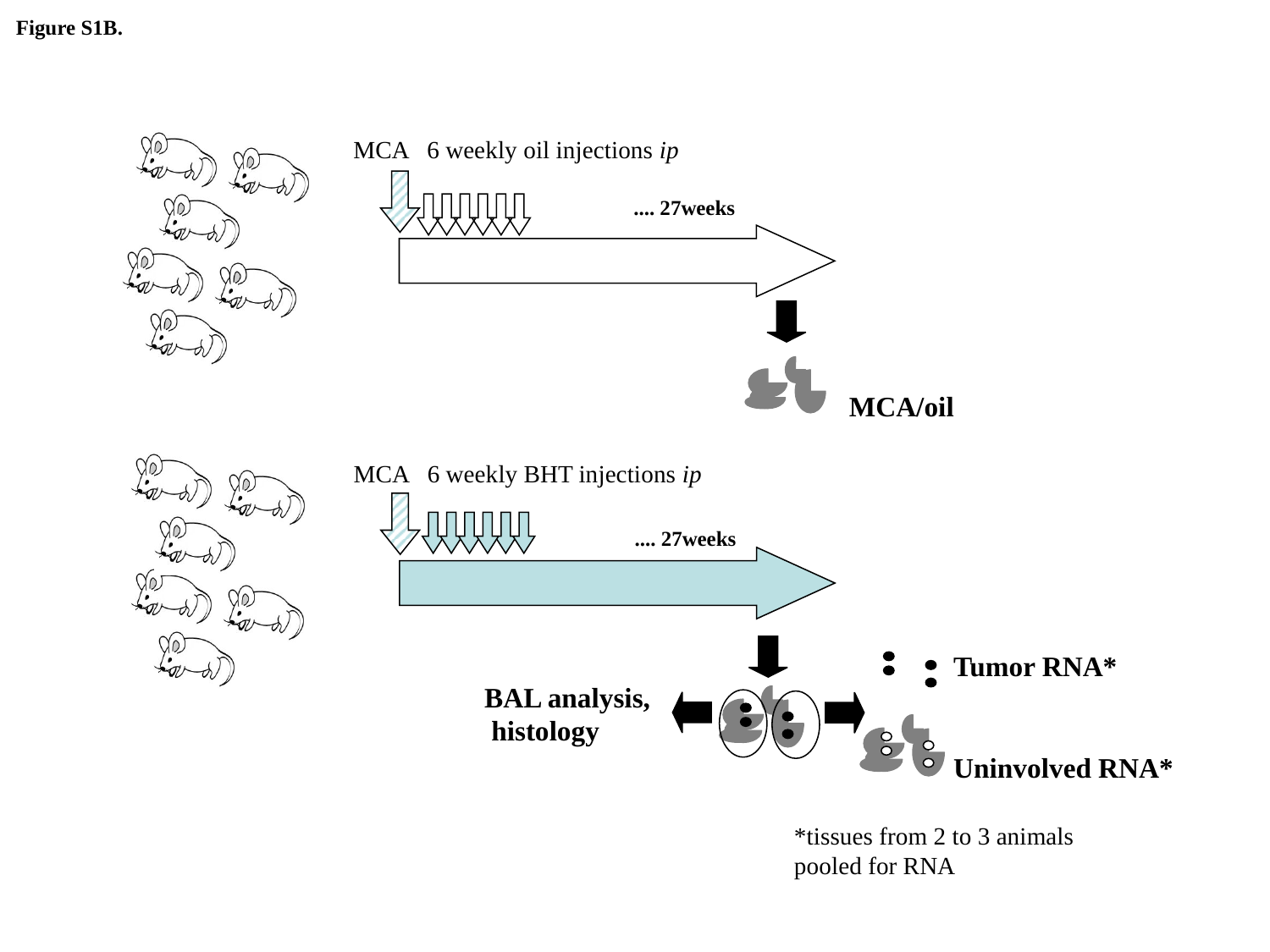

Figure S1B.
MCA
6 weekly oil injections ip
.... 27weeks
MCA/oil
MCA
6 weekly BHT injections ip
.... 27weeks
Tumor RNA*
Uninvolved RNA*
BAL analysis,
 histology
*tissues from 2 to 3 animals pooled for RNA
