# Supplementary material for: Transcriptomic analysis of pathways regulated by toll-like receptor 4 in a murine model of chronic pulmonary inflammation and carcinogenesis
Source: Mol Cancer. 2009 Nov 19;8:107. doi: 10.1186/1476-4598-8-107 (PMC2785769; doi:10.1186/1476-4598-8-107)
Supplement: Additional file 3 — Additional Methods for the Affymetrix Mouse 430A_MOE array analysis. Detailed methodology for the microarray analysis studies. [file 1476-4598-8-107-S3.DOC]

# Supplemental Methods

***Affymetrix Mouse 430A_MOE array analysis.***

Total RNA (1 µg) was amplified using the Affymetrix One-Cycle cDNA Synthesis protocol. For each array, 15 µg of amplified biotin-cRNAs was fragmented and hybridized to the array for 16 hours at 45ºC in a rotating hybridization oven using the Affymetrix Eukaryotic Target Hybridization Controls and protocol. Slides were stained with steptavidin/phycoerythrin using a double-antibody staining procedure and washed utilizing the Mini_euk2v3 Protocol of the Affymetrix Fluidics Station FS450 for antibody amplification. Arrays were scanned with an Affymetrix Scanner 3000 and data obtained using the GeneChip® Operating Software.

Because samples were hybridized to two different microarray platforms (MOE430A or MOE430Av2), data were merged based on common probeset identifiers. The CEL files for each array type (Affymetrix MOE430A or MOE430Av2) were normalized. in parallel using RMA (robust multi-array average) followed by z-transformation, or combined and normalized using singular value decomposition analysis [1, 2] followed by gc-RMA normalization [3] in order to minimize the effect of the two platforms used. Examination of principal components revealed these methods to be successful in minimizing platform effects on signal intensity.

*Supervised analysis.* The gc-RMA SVD-normalized data from Protocol 1 were loaded into GeneSpring GX 7.3.1 Expression Analysis Software (GeneSpring), transformed to linear scale and the mean intensity per gene acquired from array replicates under six experimental conditions was normalized to that in the oil (control) BALB group. A 2-way analysis of variance (ANOVA) followed by the Benjamini and Hochberg False Discovery Rate test for multiple comparisons was used to identify genes with significant (p<0.05) strain (BALB vs BALB*^Lps-d^*) vs time point (oil, 1, and 3 days following BHT) effects. Genes with greater than 2-fold change in expression compared to BALB oil controls at one or both time points were analyzed by *k*-means clustering, and 5 clusters of similar expression profiles were identified in the 476 genes; see Figure 1A for a representative pattern and Supplement Table 1A, column M, Complete GeneSpring early genes).

Data from Protocol 2 were analyzed in two ways, due to the low replicate number in the BALB tumor samples. Method 1 utilized Partek GS (St. Louis, MO) to generate ratios using the mean of the BALB MCA/oil samples as the reference, followed by two-way ANOVA with a 5% Benjamini-Hochberg False Discovery Rate test to identify genes with significant (p<0.05) strain (BALB or BALB*^Lps-d^*), treatment (MCA/oil, MCA/BHT tumor tissue, and MCA/BHT uninvolved tissue), and interaction effects. Specific contrasts were used to compare BALB tumor tissue with BALB*^Lps-d^* tumor tissue. This gene list was then imported into GeneSpring for cluster analysis (199 genes total; Supplement Materials Table 1A, column N, Complete GeneSpring late genes). In Method 2, gene lists were also generated for uninvolved tissues using Partek and the method employed for analysis of Protocol 1 samples (see Supplement Table 1A).

*Unsupervised analysis*. RMA- and z-transformed data from Protocols 1 and 2 were imported into Spotfire Decision Site (Spotfire, Somerville, MA) and analyzed separately by k-means clustering with k= 25 (early time points) or k=9 (late time points). The clusters were evaluated for biological interest, e.g. groups of profiles with changes in expression between various treatments and genetic effects which were also consistent across replicates. The centroid profile of each interesting cluster was used as a seed for similarity ranking of all profiles within the data set. Profiles similar to the seed profile were selected using an empirical cutoff in similarity score, based upon correlation with the seed profile. The sets of profiles selected for further analysis are shown in Supplement Material Table 1A, columns B, C, F, G, H and L.

Supplement Materials Table 1A contains a list of all the probes on the 430A microarray with annotation indicating cluster assignments for each probe and external IDs, gene description, GO annotation, and other information from the NetAffx site (http://www.affymetrix.com/analysis/index.affx). We identified a number of profiles of interest, and rather than provide individual gene lists we provide Supplement Materials Table 1A which has a flag column for each profile described in this paper. The flag column allows all the genes in that profile to be retrieved, together with their annotation. In addition, the Excel file permits the user to explore overlap among gene lists by selecting genes using one or more flag columns. The Legend to Supplement Materials provides a list of columns and a description of their contents. Flag columns B, C, F, G, H and L correspond to profiles found by unsupervised analysis, while columns D, E, I, J, and K correspond to profiles identified using supervised analysis. Both methods detected similar expression patterns; for instance both detected profiles which increased in samples taken 1 day following BHT treatment (profile 1) and which had a larger increase in BALB*^Lps-d^* mice (Figure 2).

The genes from the three patterns from Protocol 1 and the three common patterns from Protocol 2 were combined and analyzed using the functional classification available at DAVID (**D**atabase for **A**nnotation, **V**isualization and **I**ntegrated **D**iscovery http://david.abcc.ncifcrf.gov/; GO January 2008 version) in order to understand the total biological response to the experimental model. The output of this analysis is also provided (Supplement Materials Table 1C, early genes with GO categories; 1D late genes with GO categories). The genes in pathways with enrichment *p*-value below 3 X 10^-4^ (Protocol 1) and 1 X 10^-4^ (Protocol 2) were selected for further study (http://david.abcc.ncifcrf.gov/). Non-specific categories (defined as those with more than 100 genes, for example, “intrinsic to membrane”) were not included. Selected genes were annotated with their Gene Ontology (GO) Biological Process, and grouped to combine related categories within this sample (e.g. apoptosis and cell death were combined). This process resulted in 15 categories (Supplement Materials Table 1B). Three categories (metabolism, multicellular organismal development and signal transduction) were not numerically different between Protocols 1 and 2 and so were considered to contribute to the response under promotion and progression conditions. The distribution of transcripts in the remaining 12 categories and profiles are shown in Figure 3C.

**References**

1. Alter O, Brown PO, Botstein D: **Singular value decomposition for genome-wide expression data processing and modeling.** *Proc Natl Acad Sci U S A* 2000, **97:**10101-10106.

2. Nielsen TO, West RB, Linn SC, Alter O, Knowling MA, O'Connell JX, Zhu S, Fero M, Sherlock G, Pollack JR, et al: **Molecular characterization of soft tissue tumours: a gene expression study.** *Lancet* 2002, **359:**1301-1307.

3. Wu Z, Irizarry RA, Gentleman R, F. M-M, Spencer F: **A Model Based Background Adjustment for Oligonucleotide Expression Arrays.** *Journal of the American Statistical Association* 2004, **99:**909-917.
