# Supplementary material for: Transcriptomic analysis of pathways regulated by toll-like receptor 4 in a murine model of chronic pulmonary inflammation and carcinogenesis
Source: Mol Cancer. 2009 Nov 19;8:107. doi: 10.1186/1476-4598-8-107 (PMC2785769; doi:10.1186/1476-4598-8-107)
Supplement: Additional file 4 — Additional Table 2. Primer pairs used for the qRT-PCR analysis as described in methods, for Figs. 2 and 5. [file 1476-4598-8-107-S4.DOC]

**Table S2: Primer sequences for qRTPCR analysis.**

| **Name** |  | **Primer Sequence** | **Genbank**  **Accession no.** |
| --- | --- | --- | --- |
| Arg1 | Forward (5’🡪3’) | TTA GAG ATT ATC GGA GCG CCT | NM_007482 |
|  | Reverse (5’🡪3’) | AAG GAG CTG TCA TTA GGG ACA |  |
| Ccl17 | Forward (5’🡪3’) | GTTCCAGGGATGCCATCGTGT | NM_011332 |
|  | Reverse (5’🡪3’) | GCTGGTCACAGGCCGTTTTATG |  |
| Cfi | Forward (5’🡪3’) | TGGCATTTATATCGGTGGCTGT | NM_007686 |
|  | Reverse (5’🡪3’) | TCATTTTGGAAGGTGGCTCCAT |  |
| Cldn2 | Forward (5’🡪3’) | TTCCTATGTTGGTGCCAGCATT | NM_016675 |
|  | Reverse (5’🡪3’) | TACAAGCCAGCGAGGACATTG |  |
| *Col18a1* | Forward (5’🡪3’) | CCCATCCTCAGGTTTGTCTCTCA | D17546 |
|  | Reverse (5’🡪3’) | CTTTGATTGGCCACTTGTGACC |  |
| *Ereg* | Forward (5’🡪3’) | ATGCATCCCAGGAGAATCCGAG | NM_007950 |
|  | Reverse (5’🡪3’) | CCAGTGTAGCCCACTTCACATCTG |  |
| Fosl1 | Forward (5’🡪3’) | CTG ACA GAC TTC CTG CAG GC | U34245 |
|  | Reverse (5’🡪3’) | TGG TGC TGC CAG AAC CAC CT |  |
| Kng1 | Forward (5’🡪3’) | CGCATGGCAGGACTGTGACTT | NM_023125 |
|  | Reverse (5’🡪3’) | TCTGTCGATATGGCATGCACAC |  |
| Pparg | Forward (5’🡪3’) | GGGCGATCTTGACAGGAAAGAC | NM_011146 |
|  | Reverse (5’🡪3’) | TCTCTTGCACGGCTTCTACGG |  |
| *Pthlh* | Forward (5’🡪3’) | TGATCGCGGAGATCCACACAG | NM_008970 |
|  | Reverse (5’🡪3’) | TCTTCCCGGGTGTCTTGAGTG |  |
| *Spp1* | Forward (5’🡪3’) | CTCACCTCTCACATGAAGAGCGG | NM_009263 |
|  | Reverse (5’🡪3’) | GGCACTCTCCTGGCTCTCTTTG |  |
| *Tnc* | Forward (5’🡪3’) | AGGTTGCTGCAAACAGCAG | NM_011607 |
|  | Reverse (5’🡪3’) | CAGGAGTGGCATCAGAAAC |  |
| 18S | Forward (5’🡪3’) | GAGAAACGGCTACCACATCCAA | NR_003278 |
|  | Reverse (5’🡪3’) | CCTCCAATGGATCCTCGTTAAAG |  |
