# Supplementary figures and images for: Transcriptomic analysis of pathways regulated by toll-like receptor 4 in a murine model of chronic pulmonary inflammation and carcinogenesis
Source: Mol Cancer. 2009 Nov 19;8:107. doi: 10.1186/1476-4598-8-107 (PMC2785769; doi:10.1186/1476-4598-8-107)

## Slide 1
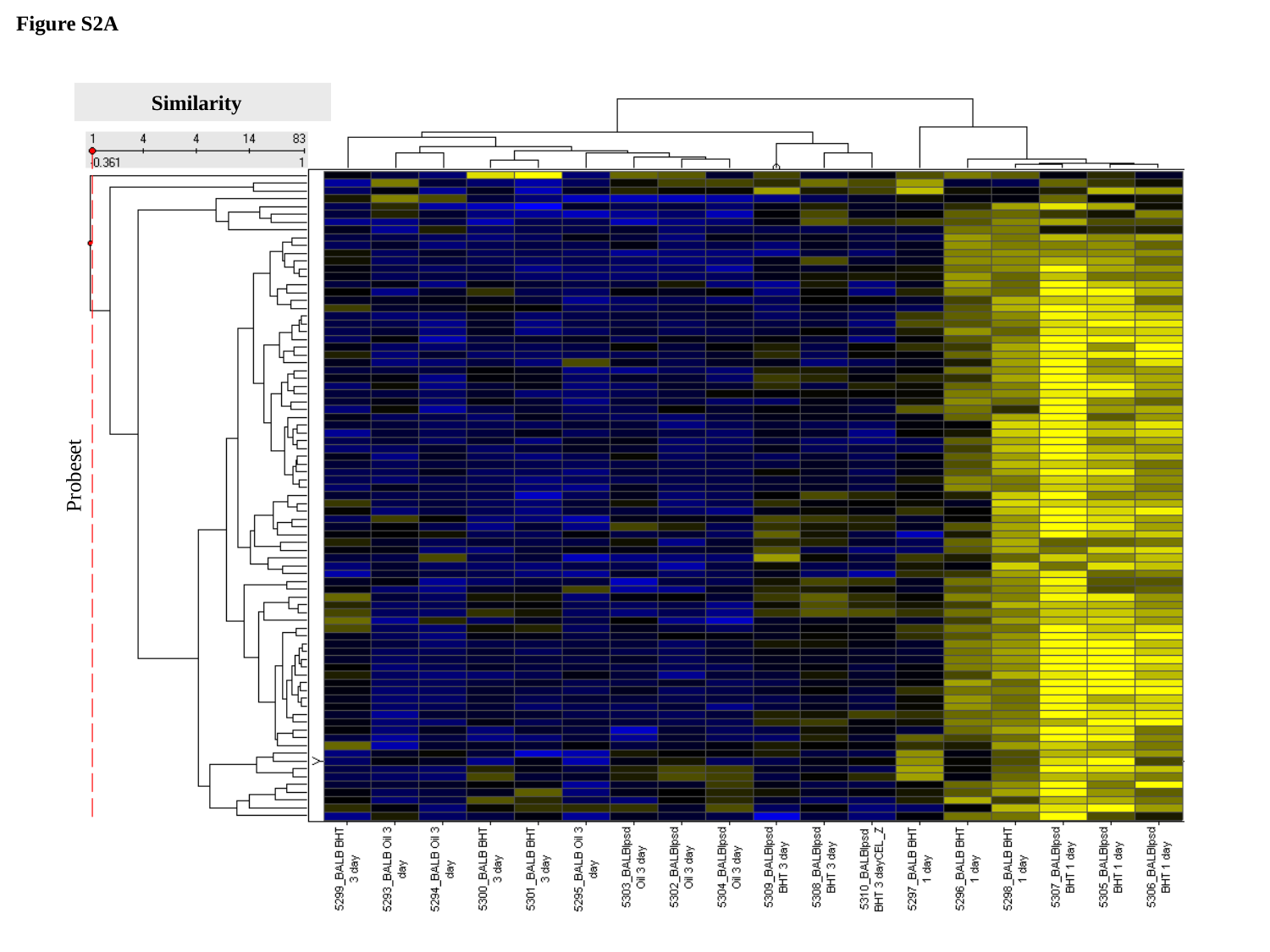

Figure S2A
Similarity
Probeset

## Slide 2
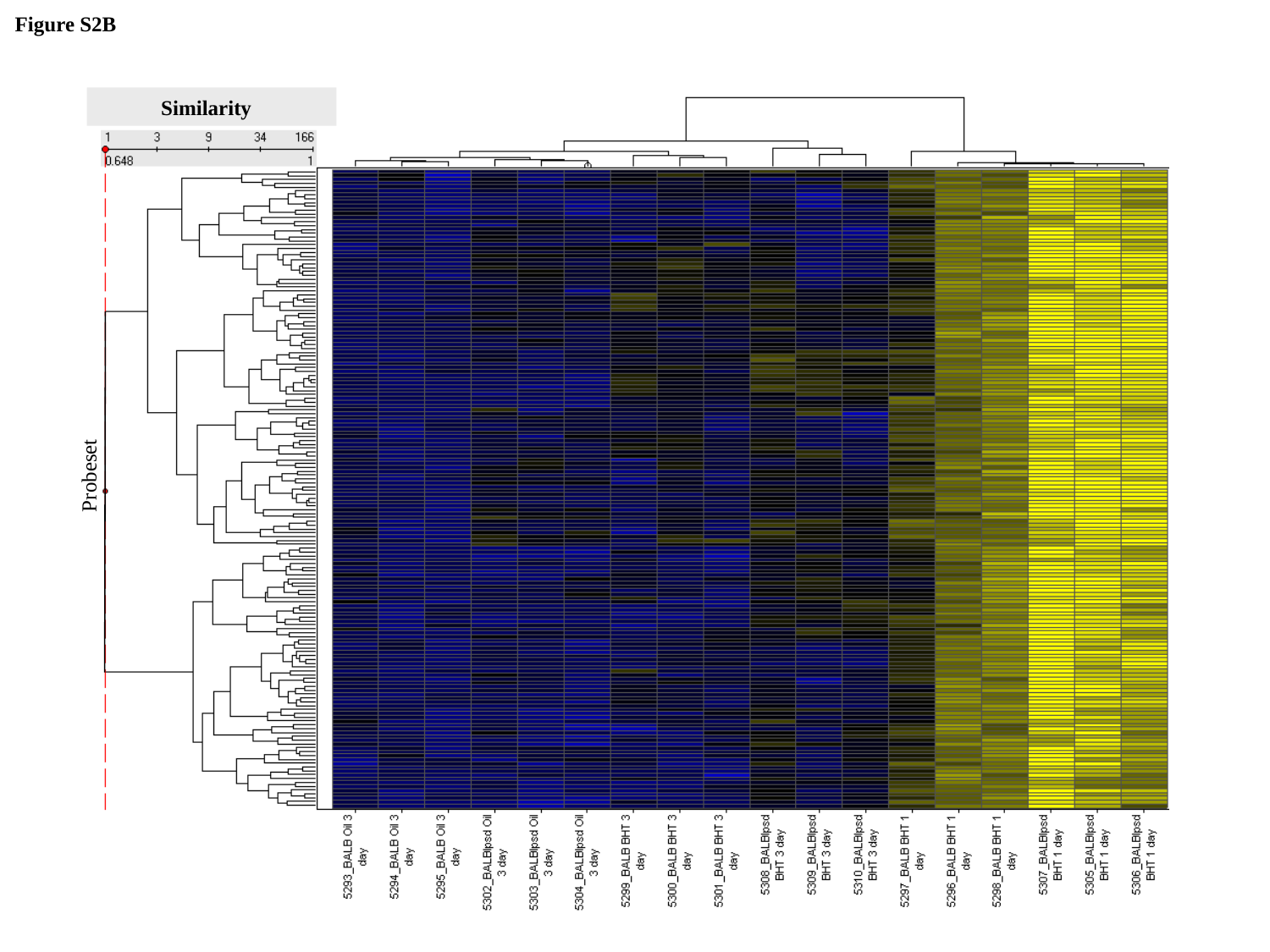

Figure S2B
Similarity
Probeset

Supplement: Additional file 5 — Additional Figure 2: Heat maps of the same representative gene lists identified for protocol 1 depicted in Figure 1. A) Expression pattern for cluster 3 (BHT_1day up_KO) identified from supervised analysis in chronic BHT-treated BALB and BALBLpsd mice over a time course (oil, 1 and 3 days following BHT). Each column represents an individual animal. From left to right, columns 2,3,6 = BALB, oil; columns 1,4,5 = BALB, 3 dy BHT; columns 13-15 = BALB, 1 dy BHT; columns 7-9 = BALBLpsd, oil; columns 10-12 = BALBLpsd, 3 dy BHT; columns 16-18 = BALBLpsd, 1 dy BHT. B) Unsupervised analysis resulting in similar expression patterns to that identified in (A). From left to right, columns 1-3 = BALB, oil; columns 4-6 = BALBLpsd, oil; columns 7-9 = BALB, 3 dy BHT; columns 10-12 = BALBLpsd, 3 dy BHT; columns 13-15 = BALB, 1 dy BHT; columns 16-18 = BALBLpsd, 1 dy BHT. N = 3 per treatment group for each strain. Y-axis is the probeset. [file 1476-4598-8-107-S5.PPT]

## Slide 1
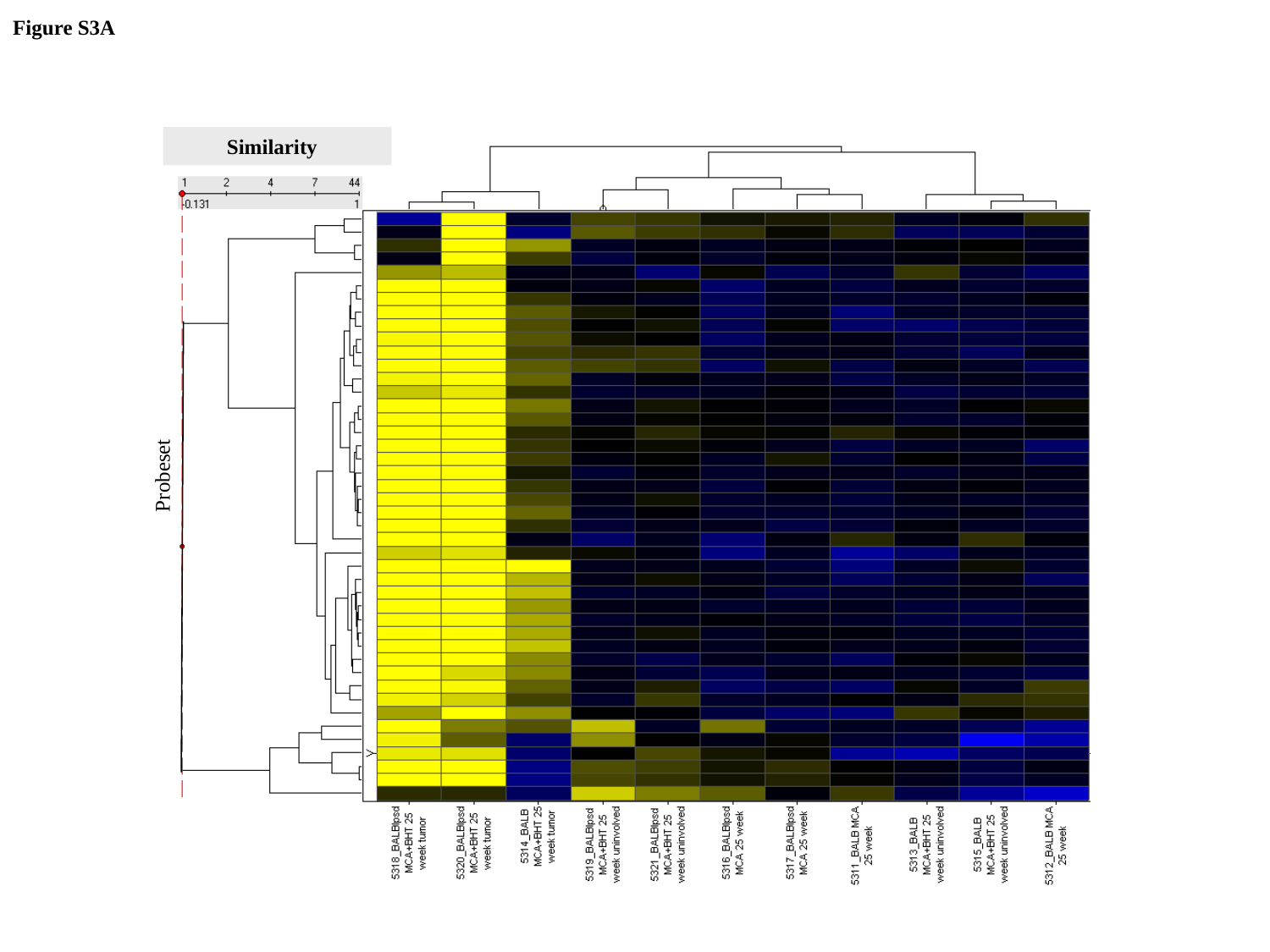

Figure S3A
Similarity
Probeset

## Slide 2
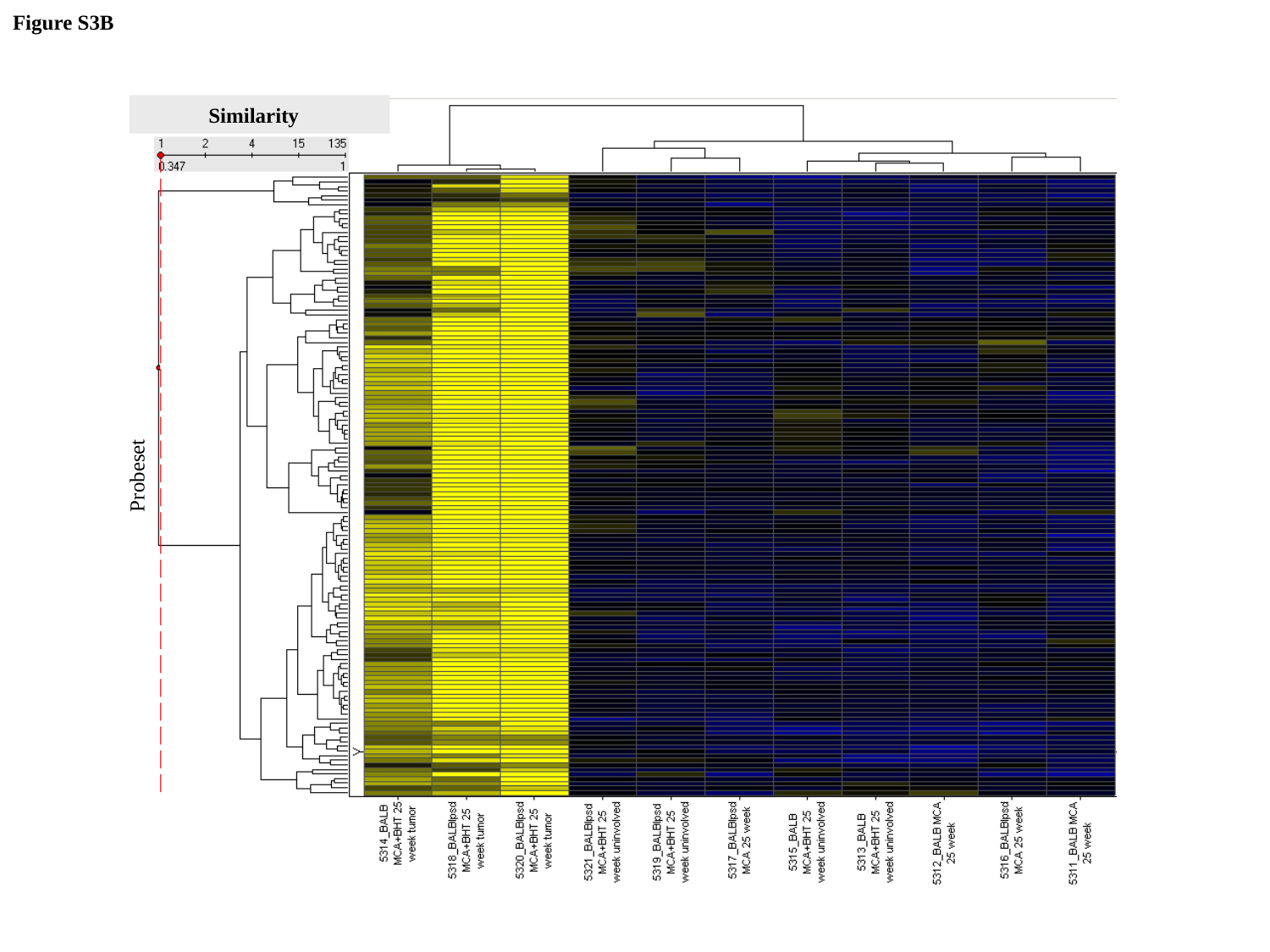

Figure S3B
Similarity
Probeset

Supplement: Additional file 6 — Additional Figure 3: Heat maps of the same representative gene lists identified from protocol 2 depicted in Figure 4. A) Expression pattern for cluster 4 (Up_KO_tumor) identified by supervised analysis. From left to right, columns 1-2 = BALBLspd, MCA/BHT-induced tumor tissue; column 3 = BALB, MCA/BHT-induced tumor tissue; columns 4-5 = BALBLspd, MCA/BHT-induced uninvolved tissue; columns 6-7 = BALBLspd, MCA exposed tissue; columns 8, 11 = BALB, MCA exposed tissue; columns 9-10 = BALB, MCA/BHT-induced uninvolved tissue. B) Unsupervised analysis with similar gene expression patterns to that observed in (A). From left to right, column 1 = BALB, MCA/BHT-induced tumor tissue; columns 2-3 = BALBLspd, MCA/BHT-induced tumor tissue; columns 4-5, BALBLspd, MCA/BHT-induced uninvolved tissue; columns 6,10 = BALBLspd, MCA exposed tissue; columns 7-8, BALB, MCA/BHT-induced uninvolved tissue; columns 10-11 = BALB, MCA exposed tissue. N = 2 per treatment group, except BALB tumor (n = 1). Y-axis is the probeset. [file 1476-4598-8-107-S6.PPT]
